# Burden of disease attributable to suboptimal diet, metabolic risks and low physical activity in Ethiopia and comparison with Eastern sub-Saharan African countries, 1990–2015: findings from the Global Burden of Disease Study 2015

**DOI:** 10.1186/s12889-018-5438-1

**Published:** 2018-04-25

**Authors:** Yohannes Adama Melaku, Molla Mesele Wassie, Tiffany K. Gill, Shao Jia Zhou, Gizachew Assefa Tessema, Azmeraw T. Amare, Yihunie Lakew, Abiy Hiruye, Tesfaye Hailu Bekele, Amare Worku, Oumer Seid, Kedir Endris, Ferew Lemma, Fisaha Haile Tesfay, Biruck Desalegn Yirsaw, Kebede Deribe, Robert Adams, Zumin Shi, Awoke Misganaw, Amare Deribew

**Affiliations:** 1 Department of Human Nutrition, Institute of Public Health, The University of Gondar, Gondar, Ethiopia; 2 Adelaide Medical School, The University of Adelaide, Adelaide, Australia; 3 School of Agriculture, Food and Wine, Faculty of Sciences, University of Adelaide, Adelaide, Australia; 4 Department of Reproductive Health, Institute of Public Health, University of Gondar, Gondar, Ethiopia; 5 School of Public Health, The University of Adelaide, Adelaide, Australia; 6 Discipline of Psychiatry, School of Medicine, The University of Adelaide, Adelaide, Australia; 7 School of Medicine and Health Sciences, Bahir Dar University, Bahir Dar, Ethiopia; 8 Department of Epidemiology, University Medical Center Groningen, the University of Groningen, Groningen, The Netherlands; 9 Ethiopian Public Health Association, Addis Ababa, Ethiopia; 10 Federal Ministry of Health, Addis Ababa, Ethiopia; 11 Food Science and Nutrition Research Directorate, Ethiopian Public Health Institue, Addis Ababa, Ethiopia; 12 Department of Public Health Sciences, Addis Continental Institute of Public Health, Addis Ababa, Ethiopia; 13 Department of Nutrition and Dietetics, School of Public Health, Mekelle University, Mekelle, Ethiopia; 14 Department of Epidemiology and Biostatics, School of Public Health, Mekelle University, Mekelle, Ethiopia; 15 Flinders University, Southgate Institute for Health, Society and Equity, Adelaide, Australia; 16 The University of South Australia, Adelaide, SA Australia; 17 Wellcome Trust Brighton and Sussex Centre for Global Health Research, Brighton and Sussex Medical School, Brighton, BN1 9PX UK; 18 School of Public Health, Addis Ababa University, Addis Ababa, Ethiopia; 19 Health Observatory, Discipline of Medicine, The Queen Elizabeth Hospital, The University of Adelaide, Adelaide, Australia; 20 Human Nutrition Department, College of Health Sciences, Qatar University, Doha, Qatar; 21 Institute of Health Metrics and Evaluation, University of Washington, Seattle, USA; 22 Nutrition International, Addis Ababa, Ethiopia; 23 St. Paul Millennium Medical College, Addis Ababa, Ethiopia

**Keywords:** Child and maternal undernutrition, Dietary risks, Metabolic risks, Physical activity, Global Burden of Disease, Ethiopia

## Abstract

**Background:**

Twelve of the 17 Sustainable Development Goals (SDGs) are related to malnutrition (both under- and overnutrition), other behavioral, and metabolic risk factors. However, comparative evidence on the impact of behavioral and metabolic risk factors on disease burden is limited in sub-Saharan Africa (SSA), including Ethiopia. Using data from the Global Burden of Disease (GBD) Study, we assessed mortality and disability-adjusted life years (DALYs) attributable to child and maternal undernutrition (CMU), dietary risks, metabolic risks and low physical activity for Ethiopia. The results were compared with 14 other Eastern SSA countries.

**Methods:**

Databases from GBD 2015, that consist of data from 1990 to 2015, were used. A comparative risk assessment approach was utilized to estimate the burden of disease attributable to CMU, dietary risks, metabolic risks and low physical activity. Exposure levels of the risk factors were estimated using spatiotemporal Gaussian process regression (ST-GPR) and Bayesian meta-regression models.

**Results:**

In 2015, there were 58,783 [95% uncertainty interval (UI): 43,653–76,020] or 8.9% [95% UI: 6.1–12.5] estimated all-cause deaths attributable to CMU, 66,269 [95% UI: 39,367–106,512] or 9.7% [95% UI: 7.4–12.3] to dietary risks, 105,057 [95% UI: 66,167–157,071] or 15.4% [95% UI: 12.8–17.6] to metabolic risks and 5808 [95% UI: 3449–9359] or 0.9% [95% UI: 0.6–1.1] to low physical activity in Ethiopia. While the age-adjusted proportion of all-cause mortality attributable to CMU decreased significantly between 1990 and 2015, it increased from 10.8% [95% UI: 8.8–13.3] to 14.5% [95% UI: 11.7–18.0] for dietary risks and from 17.0% [95% UI: 15.4–18.7] to 24.2% [95% UI: 22.2–26.1] for metabolic risks. In 2015, Ethiopia ranked among the top four countries (of 15 Eastern SSA countries) in terms of mortality and DALYs based on the age-standardized proportion of disease attributable to dietary and metabolic risks.

**Conclusions:**

In Ethiopia, while there was a decline in mortality and DALYs attributable to CMU over the last two and half decades, the burden attributable to dietary and metabolic risks have increased during the same period. Lifestyle and metabolic risks of NCDs require more attention by the primary health care system of the country.

**Electronic supplementary material:**

The online version of this article (10.1186/s12889-018-5438-1) contains supplementary material, which is available to authorized users.

## Background

Overall prevalence and burden of disease related to undernutrition have declined worldwide [1, 2], with a prediction of further success in reducing the health problem in regions and nations with the high burden, such as sub-Saharan African (SSA) countries [3]. At the same time, the burden of non-communicable diseases (NCDs) and their behavioral and metabolic risks (MRs) have increased [1, 2, 4–7]. Particularly, in developing countries, despite significant progress in tackling undernutrition and associated communicable, maternal, neonatal and nutritional diseases (CMNNDs), the burden of NCDs due to a range of health risk factors has increased [2, 6]. On top of the health sequela associated with child and maternal undernutrition (CMU) in low-income countries (LICs), dietary risks (DRs), low physical activity (LPA) and MRs have contributed to the growing burden of NCDs [4, 8–10]. Evidence also shows CMU in early life can potentially contribute to the burden of NCDs [11–16]. The double burden of malnutrition (over- and undernutrition) with a unique and unusual (non-classical) pattern of epidemiological transition (persistent high burden of CMNNDs despite the emerging burden of NCDs) [17–22] is the growing and unprecedented challenge for these countries.

Global initiatives have already recognized this phenomenon in LICs, and efforts have been geared to tackle associated factors and challenges [17]. For instance, the United Nations General Assembly labeled the decade 2015–2025 as the “Decade of Action on Nutrition” with the aim of improving overall human nutrition [23]. Moreover, 12 of the 17 Sustainable Development Goals (SDGs) are also related to malnutrition (both under- and overnutrition) and other behavioral risk factors. In particular, the second and third SDGs recognized hunger (undernutrition) and NCDs as major global challenges [24].

Ethiopia has implemented successful national programs such as primary health care which have led to tremendous public health results, including reduced child mortality [25] and increased life expectancy [6]. These achievements were mainly because of the high priority that the Ethiopian government has placed on its health policies and programs for CMNNDs, with increased investments in these areas [26, 27]. On the other hand, NCDs have been largely ignored in national programs and strategies despite the existing evidence that shows the high and increasing burden of NCDs in the country [20, 28]. Recently, in line with the global initiatives [23, 24], the government of Ethiopia has recognized the importance of addressing communicable diseases, nutritional deficiencies, maternal and neonatal disorders, and risk factors for NCDs simultaneously [27]. Particularly, efforts have been focused on reducing the burden of disease associated with major health risk factors, such as undernutrition, lifestyle and the MR factors of NCDs [27, 29]. However, limited and unreliable data on these risk factors have been a longstanding challenge to measure the performance of previous programs [26, 30, 31] and to establish a baseline for future interventions in Ethiopia [27]. In particular, the relative contribution and impact of undernutrition, lifestyle and MR factors on the current disease burden in the country have not been investigated. The Global Burden of Disease 2015 databases provide an ideal platform and opportunity to assess the burden of these factors.

In this study, we aimed to assess the mortality and disability-adjusted life years (DALYs) attributed to CMU, DRs, MRs and LPA in Ethiopia using data from the GBD Study [32]. In addition, we investigated the trend of the burden attributable to these risk factors between 1990 and 2015 in Ethiopia compared with the other 14 Eastern SSA countries. Our study will help to understand the current burden of disease attributable to the risk factors and evaluate the progress of Ethiopia in addressing these risks compared to countries in the region. Findings can also serve as baseline data for planning future interventions which will further contribute to health and health-related policies and decision making in the country.

## Methods

### Study overview

GBD is an international collaborative effort that coordinates resources, including data and expertise, to collate and disseminate health and health-related evidence at global, region, sub-region, national and sub-national levels. It uses comprehensive data sources and rigorous analysis methods to estimate the burden of disease and the risk factors [2, 6, 33]. In this study, we use GBD 2015 databases that include estimates of disease burden and risk factors from 1990 to 2015 [32].

Details of data sources and collation and computation process for estimating GBD 2015 risk factors are published elsewhere [2]. The GBD uses Guidelines for Accurate and Transparent Health Estimates Reporting (GATHER), a newly developed tool [34], to report methods and results. The GBD study also utilizes a comparative risk assessment (CRA) approach which is based on a causal framework and hierarchy of risk factors. CRA is an important analytical approach to gather data on risk factors and to estimate their relative contribution to a disease burden [35]. Within the CRA framework, risk factors are organized into four hierarchies (levels 1 to 4) [35]. In GBD 2015, 79 granular and specific risks (level 4) were classified into three major risk aggregates (behavioral, environmental/occupational and MR), which are at level 1 [2]. Risk-disease pairs with convincing or probable evidence were included as GBD 2015 risk factors [2, 36].

In our study, estimates of deaths and DALYs attributable to three behavioral risks (CMU, DRs and LPA) and five MR factors (high systolic blood pressure (SBP), high fasting plasma glucose (FPG) level, high body mass index (BMI), high total cholesterol and impaired kidney function), by sex and age for Ethiopia from 1990 to 2015, are presented. Childhood wasting, underweight and stunting, non-exclusive breast feeding, discontinued breast feeding, and iron, vitamin A, and zinc deficiencies were included under CMU. DR factors (of NCDs) included diets low in fruits, vegetables, whole grains, nuts and seeds, seafood omega-3 fatty acids, calcium, milk, fiber, polyunsaturated fatty acids, and high in sodium, processed meat, *trans* fatty acids, sugar-sweetened beverages and red meat. The risk factors were defined using the GBD’s definition [2], and their definitions are shown in Additional file 1: Table S1.

We compared the burden of disease attributable to CMU, DRs, MRs and LPA with the other 14 Eastern SSA countries based on the GBD geographical classification (Burundi, Comoros, Djibouti, Eritrea, Kenya, Madagascar, Malawi, Mozambique, Rwanda, Somalia, South Sudan, Tanzania, Uganda, and Zambia). Countries were ranked from highest (first) to lowest (15^th^) based on age-standardized population attributable fraction (PAF) of the risk factors for all-cause of deaths and DALYs. The PAF refers to the proportion of disease burden (mortality or DALYs) attributable to the risk factors [2].

### Data sources and exposure levels

Data sources used for each country in the Eastern SSA countries can be accessed on the Global Health Data Exchange (http://ghdx.healthdata.org/). Additional file 1: Table S2 provides data sources used to estimate exposure levels of CMU, DRs, MRs and LPA for Ethiopia. For CMU, data were collated from various sources, including demographic and health surveys, and the Food and Agriculture (FAO) Food Balance Sheet, and United Nations International Children’s Emergency Fund (UNICEF) and World Health Organization (WHO) databases [2].

Multiple data sources, including the FAO Food Balance Sheet and household budget surveys, were used to estimate exposure levels of DRs of NCDs. For *trans* fatty acids, availability of partially hydrogenated vegetable oil packaged foods was used. All DR factors were standardized to 2000 kcal/day except urinary sodium and sugar-sweetened beverages. The methods for estimating the burden of disease related to DRs in Ethiopia have been published previously [37]. For MRs, data were collated from sources, including surveys, longitudinal studies, published literature which provided both measured or self-reported MRs. For LPA, the WHO’s non-communicable disease risk factor surveys were used [2].

Two main modeling strategies were used to estimate the exposure levels of risk factors: 1) a spatiotemporal Gaussian process regression model (ST-GPR) and; 2) a Bayesian meta-regression model (DisMod-MR 2.1) which are mixed effect models that borrow information across geographies (global, super-region, region, nation and subnational), age, sex, and time. These approaches allow for the pooling of data from different sources and adjustment of bias.

Covariates that potentially effect the intake level of individual DR factors were incorporated to assist in the predictions for locations and time where there is a lack of data. For instance, being landlocked (yes/no) was used to estimate intake level of seafood omega-3 fatty acids. Adjustments, including age-sex splitting, adding study level covariates, and bias correction for all risk factors were performed [2]. Study level covariates that could potentially impact the estimates of dietary exposure, such as dietary data collection methods (i.e., 24-h diet recall, food frequency questionnaire, household budget surveys, or FAO Food Balance Sheets), were considered in the model. Country and study level covariates used in the modeling of DRs are shown in Additional file 1: Table S3.

### Relative risks

Relative risks of risk-disease pairs were obtained from meta-analyses of prospective observational studies or randomized controlled trials. The GBD 2015 risk factors paper contains detailed methods on how the relative risks of each of the risk factors were estimated [2]. Metabolic mediators (through which a risk factor may have an effect) of DRs are provided in Additional file 1: Table S3.

### Attributable mortality and DALYs and uncertainties

The proportion of mortality and DALYs that could have been prevented if the exposure level of a risk factor had been sustained at the level associated with the lowest risk was calculated. The level of exposure that is associated with the lowest risk is called theoretical minimum-risk exposure level (TMREL). A 20% uncertainty range below and above the TMREL was applied (Additional file 1: Table S1).

To determine the mortality and DALYs attributable to the risk factors, the PAF was firstly determined using the following inputs: the exposure level for each risk factor: relative risks, TMREL, and the total number of deaths from the specific disease. Using the Monte Carlo approach, the uncertainty of parameters for exposure, relative risk, attributable mortality and DALYs [summing up years lived with disability and years of life lost] were calculated with 1000 repeated draws. Detailed formulas and computation approaches are provided elsewhere [2, 6, 33].

In this study, both crude and adjusted estimates of deaths and DALYs attributed to CMU, DRs, MRs and LPA are provided. The GBD world population standard was used for the computation of age-standardized estimates. Results are presented as means with 95% uncertainty intervals (UI) in parenthesis. We calculated percentage change on the basis of the point estimates.

## Results

### Burden of disease attributable to CMU, DRs, MRs and LPA in 2015, Ethiopia (crude estimates)

In 2015, there were 58,783 [43,653–76,020], 66,269 [39,367–106,512], 105,057 [66,167–157,071] and 5808 [3449–9359] estimated deaths attributable to CMU, DRs, MR and LPA in both sexes in Ethiopia, respectively. Of all deaths, 8.9% [6.1–12.5], 9.7% [7.4–12.3], 15.4% [12.8–17.6] and 0.9% [0.6–1.1] were attributable to CMU, DRs, MRs and LPA, respectively. These represent 17.7% [12.8–23.0] deaths due to CMNND, 23.1% [18.6–28.5], 34.3% [31.3–37.3] and 2.0% [1.4–2.7] due to NCD deaths, respectively. CMU, DRs, MRs and LPA were associated with 5,312,975 [4,068,319–6,720,367], 1,698,099 [1,026,366–2,736,469], 2,706,312 [1,755,853–4,005,055] and 140,484 [84,760–224,637] estimated DALYs, representing 23.2% [17.5–29.0] of CMNND, and 11.3% [8.1–15.2], 16.9% [13.5–20.7] and 0.9% [0.6–1.3] NCD DALYs in 2015 in the country, respectively (Table 1).

**Table 1:** Number, crude rate and proportion (95% uncertainty interval) of deaths and disability-adjusted life years attributable to child and maternal undernutrition, low physical activity, dietary and metabolic risk factors in Ethiopia, 2015

Risk factors	Causes	Metric	Both sexes	Males	Females
			Deaths		
Child and maternal undernutrition	All causes	Number	58,783 (43,653–76,020)	31,016 (22,624–40,885)	27,768 (19,541–37,070)
		Rate per 100,000	59 (44–76)	62 (46–82)	56 (39–74)
		Proportion (%)	8.9% (6.1–12.5)	9% (5.5–13.4)	9.1% (5.5–14.1)
	Communicable, maternal, neonatal, and nutritional diseases	Proportion (%)	17.7% (12.8–23.0)	17.9% (12.5–24.3)	17.7% (11.9–24.4)
Dietary risks	All causes	Number	66,269 (39,367–106,512)	35,122 (19,540–66,193)	31,147 (14,959–58,347)
		Rate per 100,000	67 (40–107)	71 (39–133)	63 (30–117)
		Proportion (%)	9.7% (7.4–12.3)	9.5% (7.2–12.8)	9.6% (6.9–13.0)
	Non-communicable diseases	Proportion (%)	23.1% (18.6–28.5)	23.9% (19.1–29.4)	22.1% (17.2–27.9)
Metabolic risks	All causes	Number	105,057 (66,167–157,071)	52,270 (30,895–93,837)	52,787 (26,896–94,624)
		Rate per 100,000	106 (67–158)	105 (62–189)	106 (54–190)
		Proportion (%)	15.4% (12.8–17.6)	14.2% (11.6–17.1)	16.3% (12.8–19.4)
	Non-communicable diseases	Proportion (%)	34.3% (31.3–37.3)	33.2% (29.9–36.6)	35.3% (31.5–39.0)
	Communicable, maternal, neonatal, and nutritional diseases	Proportion (%)	0.8% (0.5–1.4)	1.0% (0.5–1.9)	0.6% (0.3–1.0)
Low physical activity	All causes	Number	5808 (3449–9359)	3640 (1982–6519)	2168 (1017–4232)
		Rate per 100,000	6 (4–9)	7 (4–13)	4 (2–9)
		Proportion (%)	0.9% (0.59–1.12)	1.0% (0.7–1.3)	0.7% (0.4–1.0)
	Non-communicable diseases	Proportion (%)	2.0% (1.4–2.7)	2.5% (1.8–3.2)	1.5% (1.0–2.1)
			**Disability-adjusted life years**		
Child and maternal undernutrition	All causes	Number	5,312,975 (4,068,319–6,720,367)	2,835,570 (2,124,837–3,684,776)	2,477,404 (1,813,335–3,274,603)
		Rate per 100,000	5343 (4092–6759)	5713 (4281–7424)	4975 (3641–6576)
		Proportion (%)	13.0% (9.7–16.8)	13.0% (9.1–17.7)	13.2% (9.0–18.0)
	Communicable, maternal, neonatal, and nutritional diseases	Proportion (%)	23.2% (17.5–29.0)	23.4% (17.4–29.8)	23.1% (16.5–30.0)
Dietary risks	All causes	Number	1,698,099 (1,026,366–2,736,469)	947,273 (525785–1,796,933)	750,826 (380840–1,398,001)
		Rate per 100,000	1708 (1032–2752)	1909 (1059–3620)	1508 (765–2807)
		Proportion (%)	4.1% (2.8–5.7)	4.2% (2.8–6.5)	3.9% (2.4–5.8)
	Non-communicable diseases	Proportion (%)	11.3% (8.1–15.2)	12.1% (8.4–17.3)	10.2% (6.5–14.8)
Metabolic risks	All causes	Number	2,706,312 (1,755,853–4,005,055)	1,449,061 (879,099–2,577,821)	1,257,252 (708,492–2,172,528)
		Rate per 100,000	2722 (1766–4028)	2920 (1771–5194)	2525 (1423–4363)
		Proportion (%)	6.5% (4.9–8.3)	6.4% (4.8–9.0)	6.5% (4.4–9.0)
	Non-communicable diseases	Proportion (%)	16.9% (13.5–20.7)	17.2% (13.3–22.5)	16.1% (11.5–21.3)
	Communicable, maternal, neonatal, and nutritional diseases	Proportion (%)	0.4% (0.2–0.7)	0.5% (0.2–1.0)	0.3% (0.1–0.5)
Low physical activity	All causes	Number	140,484 (84,760–224,637)	91,637 (51,409–163,588	48,847 (25,132–91,957)
		Rate per 100,000	141 (85–226)	185 (104–330)	98 (51–185)
		Proportion (%)	0.3% (0.2–0.5)	0.4% (0.3–0.6)	0.3% (0.2–0.4)
	Non-communicable diseases	Proportion (%)	0.9% (0.6–1.3)	1.2% (0.8–1.7)	0.7% (0.4–1.0)

When considering specific risk factors of CMU, childhood wasting (13.4% [8.9–18.1]), underweight, (5.2% [3.0–8.5]), non-exclusive breast feeding (3.3% [1.6–5.5]) and stunting (3.3% [1.3–6.6]) were the most common contributors of deaths due to CMNND in 2015. Of the DRs, a diet low in fruits (8.1% [5.5–10.9]), vegetable (5.2% [2.7–8]), whole grain (5.0% [3.1–7.2]), nuts and seeds (4.3% [2.7–6.3]) and high in sodium (4.5% [0.8–11.9]) were most common risks of NCD deaths. Twenty-three percent [20.4–25.8], 9.7% [8.0–11.5], 6.2% [3.6–9.4], 5.1% [3.4–7.2], and 4.1% [3.3–5.0] of NCD deaths were attributable to high SBP, high FPG, high BMI, high total cholesterol and impaired kidney function, respectively. Childhood wasting, underweight and stunting, and non-exclusive breast feeding were the major CMU contributors of CMNND DALYs. High SBP, high FPG and high BMI contributed to 9.9% (7.4–12.9), 5.9% (4.9–7.1), and 3.7% (2.1–5.8) NCD DALYs, respectively (Table 2).

**Table 2:** Crude proportion (95% uncertainty interval) of deaths and disability-adjusted life years attributable to specific types of child and maternal undernutrition, dietary and metabolic risk factors in Ethiopia, 2015

Risk factors	Proportion (%) (95% UI)			
	Deaths		Disability-adjusted life years	
	All causes	Communicable, maternal, neonatal, and nutritional diseases	All causes	Communicable, maternal, neonatal, and nutritional diseases
**Child and maternal undernutrition**	**8.9 (6.1–12.5**)	**17.7 (12.8–23)**	**13.0 (9.7–16.8)**	**23.2 (17.5–29.0)**
Childhood undernutrition	7.5 (4.8–11.0)	15.0 (10.3–20.2)	10.8 (7.7–14.4)	19.2 (14–24.7)
Childhood wasting	6.7 (4.2–9.7)	13.4 (8.9–18.1)	9.7 (6.8–12.9)	17.2 (12.2–22.3)
Childhood underweight	2.6 (1.4–4.5)	5.2 (3.0–8.5)	3.8 (2.3–6.1)	6.8 (4.2–10.6)
Childhood stunting	1.7 (0.6–3.5)	3.3 (1.3–6.6)	2.4 (1.0–4.6)	4.2 (1.7–7.9)
Suboptimal breastfeeding	1.7 (0.9–3.0)	3.5 (1.8–5.8)	2.5 (1.3–4.1)	4.4 (2.3–7.2)
Non-exclusive breastfeeding	1.6 (0.8–2.9)	3.3 (1.6–5.5)	2.3 (1.1–3.9)	4.1 (2.0–6.8)
Discontinued breastfeeding	0.1 (0.0–0.3)	0.3 (0.1–0.6)	0.2 (0.1–0.5)	0.3 (0.1–0.8)
Iron deficiency	1.0 (0.7–1.6)	2.1 (1.3–3.2)	1.8 (1.3–2.4)	3.2 (2.3–4.2)
Vitamin A deficiency	0.6 (0.2–2.0)	1.2 (0.4–3.7)	0.9 (0.3–2.6)	1.5 (0.5–4.4)
Zinc deficiency	0.2 (0.0–0.5)	0.3 (0.0–1.0)	0.2 (0.0–0.7)	0.4 (0.0–1.2)
**Metabolic risks**	**0.6 (0.5–0.7)**	**0.8 (0.5–1.4)**	**0.2 (0.2–0.2)**	**0.4 (0.2–0.7)**
High fasting plasma glucose	4.5 (3.5–5.4)	0.8 (0.5–1.4)	2.4 (1.8–2.9)	0.4 (0.2–0.7)
	**All causes**	**Non-communicable diseases**	**All causes**	**Non-communicable diseases**
**Dietary risks**	**9.7 (7.4–12.3)**	**23.1 (18.6–28.5)**	**4.1 (2.8–5.7)**	**11.3 (8.1–15.2)**
Diet low in fruits	3.4 (2.2–4.7)	8.1 (5.5–10.9)	1.5 (0.9–2.3)	4.2 (2.6–6.1)
Diet low in vegetables	2.2 (1.1–3.4)	5.2 (2.7–8.0)	0.9 (0.4–1.5)	2.5 (1.2–4.1)
Diet low in whole grains	2.1 (1.3–3.1)	5.0 (3.1–7.2)	1.0 (0.6–1.6)	2.8 (1.6–4.4)
Diet high in sodium	1.9 (0.3–4.9)	4.5 (0.8–11.9)	0.8 (0.1–2.0)	2.1 (0.4–5.5)
Diet low in nuts and seeds	1.8 (1.1–2.7)	4.3 (2.7–6.3)	0.8 (0.5–1.3)	2.2 (1.3–3.4)
Diet low in seafood omega-3 fatty acids	1.4 (0.6–2.3)	3.4 (1.4–5.4)	0.5 (0.2–1.0)	1.5 (0.6–2.7)
Diet high in processed meat	0.6 (0.2–1.0)	1.4 (0.5–2.3)	0.3 (0.1–0.5)	0.8 (0.4–1.4)
Diet high in trans fatty acids	0.4 (0.1–0.7)	0.9 (0.3–1.7)	0.2 (0.1–0.4)	0.5 (0.2–1.0)
Diet suboptimal in calcium	0.1 (0.1–0.2)	0.3 (0.2–0.5)	0.1 (0.0–0.1)	0.2 (0.1–0.2)
Diet high in sugar-sweetened beverages	0.0 (0.0–0.1)	0.1 (0.1–0.1)	0.0 (0.0–0.0)	0.1 (0.0–0.1)
Diet low in milk	0.1 (0.0–0.2)	0.2 (0.1–0.4)	0.0 (0.0–0.1)	0.1 (0.0–0.2)
Diet high in red meat	0.0 (0.0–0.0)	0.0 (0.0–0.1)	0.0 (0.0–0.0)	0.0 (0.0–0.1)
Diet low in fiber	0.0 (0.0–0.0)	0.0 (0.0–0.1)	0.0 (0.0–0.0)	0.0 (0.0–0.1)
Diet low in polyunsaturated fatty acids	0.0 (0.0–0.1)	0.1 (0.0–0.1)	0.0 (0.0–0.0)	0.0 (0.0–0.1)
**Metabolic risks**	**15.4 (12.8–17.6)**	**34.3 (31.3–37.3)**	**6.5 (4.9–8.3)**	**16.9 (13.5–20.7)**
High systolic blood pressure	9.6 (7.9–11.3)	23.1 (20.4–25.8)	3.6 (2.5–4.8)	9.9 (7.4–12.9)
High fasting plasma glucose		9.7 (8.0–11.5)		5.9 (4.9–7.1)
High body-mass index	2.6 (1.4–4.0)	6.2 (3.6–9.4)	1.4 (0.7–2.1)	3.7 (2.1–5.8)
High total cholesterol	2.1 (1.4–3.1)	5.1 (3.4–7.2)	0.9 (0.5–1.3)	2.4 (1.5–3.6)
Impaired kidney function	1.7 (1.3–2.1)	4.1 (3.3–5.0)	0.8 (0.6–1.0)	2.3 (1.8–2.8)

*UI* uncertainty interval; Zero (0.0) indicates very low proportion. The sum of percentages of risk factors in a column exceeds the total for all risk factors combined under a risk factor cluster because of an overlap between various risk factors

**Fig. 1:**
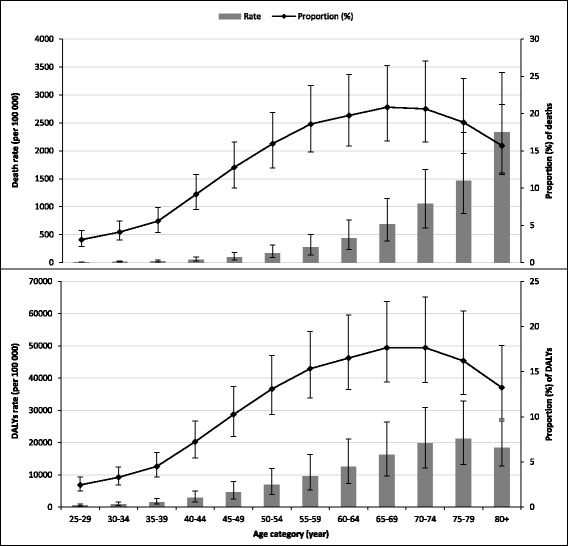
Rate and proportion (95% uncertainty interval) of deaths and disability-adjusted life years (DALYs) attributable to dietary risk factors by age in Ethiopia, 2015 (*Proportion was calculated out of all-cause of death*)

**Fig. 2:**
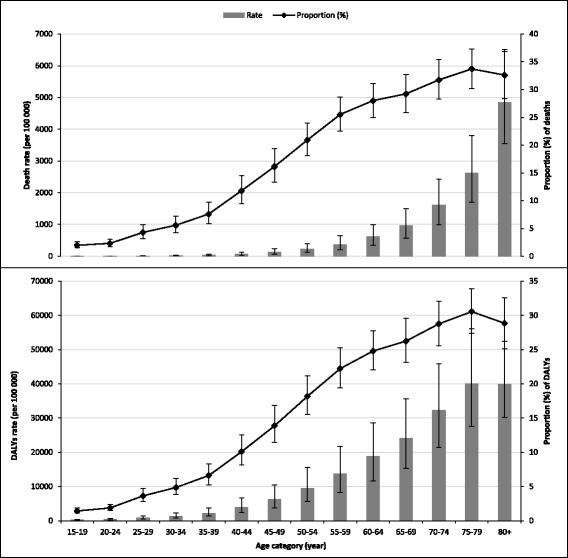
Rate and proportion (95% uncertainty interval) of deaths and disability-adjusted life years (DALYs) attributable to metabolic risk factors by age in Ethiopia, 2015 (*Proportion was calculated out of all-cause of death*)

**Fig. 3:**
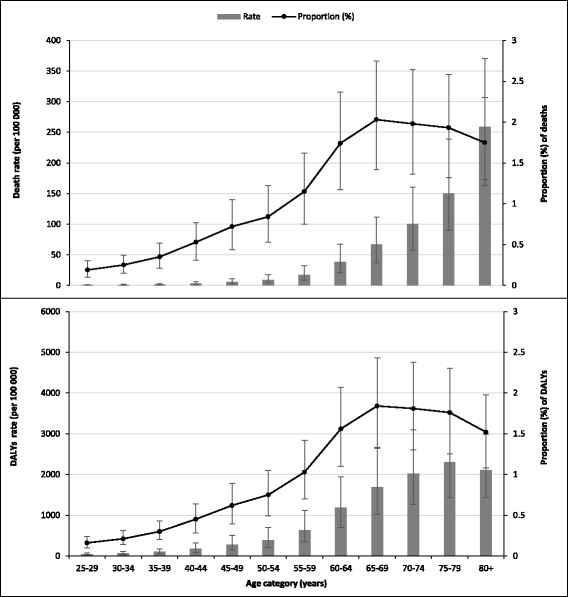
Rate and proportion (95% uncertainty interval) of deaths and disability-adjusted life years (DALYs) attributable to low physical activity by age in Ethiopia, 2015 (*Proportion was calculated out of all-cause of death*)

The pattern of mortality and DALYs attributable to DRs, MRs and LPA by age category are depicted in Figs. 1, 2, 3 and Additional file 1: Figure S1.

### Trend of disease burden attributable to CMU, DRs, MRs and LPA between 1990 and 2015, Ethiopia

Table 3 shows age-standardized death and DALY rates and proportions, and the percentage change between 1990 and 2015 in Ethiopia. The age-standardized proportion of all deaths attributable to CMU decreased by more than half over the past 25 years, from 8.6% [6.8–11.0] in 1990 to 3.6% [2.5–5.1] in 2015. Over the same period, the age-standardized death rate attributable to CMU decreased drastically from 231 [185–294] to 44 (33–56) per 100,000 people. The proportion of deaths attributable to specific types of CMU, particularly childhood wasting and underweight, also decreased. However, the proportion of all-cause deaths attributable to DRs and MRs increased from 10.8% [8.8–13.3] to 14.5% [11.7–18.0] and from 17.0% [15.4–18.7] to 24.2% [22.2–26.1], respectively. While the proportion of CMNND related deaths attributable to CMU decreased by half, from 18.4% [14.7–23.6] to 9.9% [7.3–13.4] over the past 25 years, NCD deaths attributable to DRs (from 10.8% [8.8–13.3] to 14.5% [11.7–18.0]) and MRs (from 17.0% [15.4–18.7] to 24.2% [22.2–26.1]), and LPA (from 1.4% [1.0–1.9] to 1.5% [1.0–1.9]) increased in Ethiopia (Table 3 and Additional file 1: Figures S2 and S3).

**Table 3:** Age-standardized rate, proportion and proportion of change (95% uncertainty interval) of deaths and disability-adjusted life years attributable to child and maternal undernutriton, low physical activity, dietary and metabolic risk factors in Ethiopia, 1990–2015

Risk factors	Age-standardized rate per 100,000 (95% UI)		Age-standardized proportion (%) (95% UI)			Age-standardized proportion (%) (95% UI)	
	1990	2015	1990	2015	Change in proportion (%)	1990	2015
	Deaths						
				All causes		Communicable, maternal, neonatal, and nutritional diseases	
**Child and maternal undernutrition**	**231 (185–294)**	**44 (33–56)**	**8.6 (6.8–11)**	3.6 (2.5–5.1)*	**−58.5 (−72.4 to − 39.7)**	**18.4 (14.7–23.6)**	9.9 (7.3–13.4)*
Childhood undernutrition	195 (150–254)	30 (22–40)	7.3 (5.5–9.5)	2.5 (1.5–3.9)*	−65.4 (−80.1 to − 43.5)	15.5 (11.8–20.4)	7.0 (4.4–10.3)*
Childhood wasting	162 (113–230)	27 (19–36)	6.0 (4.1–8.7)	2.2 (1.3–3.4)*	−62.8 (−78.8 to −37.4)	12.9 (8.9–18.5)	6.2 (3.9–9)*
Childhood underweight	85 (50–148)	10 (6–17)	3.2 (1.8–5.5)	0.9 (0.4–1.5)*	−72.6 (−87.4 to − 43.7)	6.8 (3.9–11.5)	2.4 (1.3–4.2)
Childhood stunting	55 (24–108)	7 (3–13)	2.0 (0.9–4.1)	0.6 (0.2–1.2)	−72.7 (−89.9 to − 41.2)	4.4 (2.0–8.7)	1.6 (0.6–3.2)
Suboptimal breastfeeding	31 (15–49)	7 (4–12)	1.1 (0.5–1.8)	0.6 (0.3–1.0)	−49.9 (− 75.6 to 3.2)	2.5 (1.2–4.0)	1.6 (0.8–2.7)
Non-exclusive breastfeeding	28 (14–45)	7 (3–11)	1.0 (0.5–1.7)	0.5 (0.2–1.0)	−47.7 (− 74.8 to 5.7)	2.2 (1.1–3.6)	1.5 (0.7–2.6)
Discontinued breastfeeding	4 (1–9)	1 (0–1)	0.1 (0.0–0.3)	0.0 (0.0–0.1)	− 68.8 (− 88.2 to 4.8)	0.3 (0.1–0.7)	0.1 (0–0.3)
Iron deficiency	30 (19–44)	12 (7–20)	1.1 (0.7–1.6)	1.0 (0.6–1.4)	− 12.3 (− 35.5 to 24.2)	2.4 (1.5–3.4)	2.7 (1.8–3.9)
Vitamin A deficiency	36 (12–86)	2 (1–7)	1.3 (0.5–3.2)	0.2 (0.1–0.7)	− 84.9 (− 96.2 to − 35.5)	2.9 (1.0–6.8)	0.6 (0.2–1.9)
Zinc deficiency	5 (0–14)	1 (0–2)	0.2 (0.0–0.5)	0.1 (0.0–0.2)	−68.5 (− 90.6 to 17.1)	0.4 (0.0–1.1)	0.2 (0.0–0.5)
**Metabolic risks**	**16 (9–25)**	**6 (3–12)**	**17.0 (15.4–18.7)**	24.2 (22.2–26.1)*	**42.5 (27.1 to 58.3)**	**1.2 (0.7–1.9)**	**1.4 (0.8–2.3)**
High fasting plasma glucose	16 (9–25)	6 (3–12)	4.5 (3.8–5.3)	6.7 (5.6–8.0)*	48.1 (23.9 to 72)	1.2 (0.7–1.9)	1.4 (0.8–2.3)
			**All causes**		**Change in proportion (%)**	**Non-communicable diseases**	
**Dietary risks**	**290 (231–364)**	**182 (112–282)**	**10.8 (8.8–13.3)**	**14.5 (11.7–18.0)**	**35.0 (14.1 to 53.9)**	**25.6 (21.4–31.3)**	**25.6 (20.6–31.6)**
Diet low in fruits	105 (71–141)	61 (33–99)	3.9 (2.7–5.3)	4.9 (3.3–6.5)	25 (2.5 to 45.3)	9.3 (6.5–12.1)	8.6 (5.8–11.4)
Diet low in vegetables	65 (35–101)	40 (17–72)	2.4 (1.3–3.7)	3.2 (1.7–4.9)	32.5 (6.3 to 55.7)	5.7 (3.1–8.8)	5.6 (3.0–8.7)
Diet low in whole grains	61 (38–88)	37 (18–66)	2.3 (1.4–3.3)	2.9 (1.8–4.3)	29.8 (4.5 to 51.3)	5.4 (3.4–7.6)	5.2 (3.3–7.5)
Diet high in sodium	58 (10–155)	35 (5–105)	2.2 (0.4–5.9)	2.8 (0.5–7.5)	28.7 (− 0.6 to 57.4)	5.2 (0.9–13.7)	4.9 (0.9–13.3)
Diet low in nuts and seeds	50 (30–72)	34 (18–59)	1.8 (1.2–2.6)	2.7 (1.7–3.9)	47.8 (19.2 to 73.0)	4.4 (2.8–6.2)	4.8 (2.9–6.9)
Diet low in seafood omega-3 fatty acids	39 (16–64)	26 (10–50)	1.4 (0.6–2.4)	2.1 (0.9–3.4)	43.5 (15.0 to 70.0)	3.4 (1.5–5.6)	3.7 (1.6–5.9)
Diet high in processed meat	16 (6–26)	10 (3–19)	0.6 (0.2–1.0)	0.8 (0.3–1.4)	36.3 (8.8 to 62.2)	1.4 (0.5–2.3)	1.4 (0.5–2.4)
Diet high in trans fatty acids	10 (3–20)	6 (2–14)	0.4 (0.1–0.7)	0.5 (0.2–1.0)	33.0 (1.4 to 61.8)	0.9 (0.3–1.7)	0.9 (0.3–1.8)
Diet low in milk	3 (1–5)	2 (1–4)	0.1 (0.0–0.2)	0.2 (0.1–0.3)	62.4 (37.3 to 95.4)	0.2 (0.1–0.4)	0.3 (0.1–0.5)
Diet suboptimal in calcium	4 (2–5)	3 (2–5)	0.1 (0.1–0.2)	0.2 (0.1–0.3)	61.2 (36.3 to 94.4)	0.3 (0.2–0.5)	0.4 (0.3–0.5)
Diet high in red meat	0 (0–1)	0 (0–0)	0.0 (0.0–0.0)	0.0 (0.0–0.0)	31.7 (2.3 to 66.5)	0.0 (0.0–0.1)	0.0 (0.0–0.1)
Diet high in sugar-sweetened beverages	1 (0–1)	1 (0–1)	0.0 (0.0–0.0)	0.0 (0.0–0.1)	51.6 (19.4 to 98.2)	0.1 (0.0–0.1)	0.1 (0.1–0.1)
Diet low in fiber	0 (0–1)	0 (0–1)	0.0 (0.0–0.0)	0.0 (0.0–0.0)	54.7 (−4.8 to 137.7)	0.0 (0.0–0.1)	0.0 (0.0–0.1)
Diet low in polyunsaturated fatty acids	1 (0–1)	1 (0–1)	0.0 (0.0–0.1)	0.0 (0.0–0.1)	42.9 (14.3 to 69.3)	0.1 (0.0–0.1)	0.1 (0.0–0.1)
**Metabolic risks**	**419 (357–483)**	**283 (185–413)**	**17.0 (15.4–18.7)**	24.2 (22.2–26.1)*	**42.5 (27.1 to 58.3)**	**37.1 (34.6–39.7)**	**39.8 (36.8–42.8)**
High systolic blood pressure	286 (238–339)	193 (124–284)	10.6 (9.1–12.2)	15.4 (13.5–17.4)*	45.5 (28.4 to 64.3)	25.3 (22.4–28.4)	27.2 (24.0–30.5)
High fasting plasma glucose	106 (86–129)	77 (49–117)	4.5 (3.8–5.3)	6.7 (5.6–8.0)*	48.1 (23.9 to 72.0)	9.4 (8.1–11.0)	10.9 (9.1–13.2)
High body-mass index	33 (14–58)	45 (21–81)	1.2 (0.5–2.2)	3.6 (2.0–5.6)	196.8 (110.6 to 367.1)	2.9 (1.3–5.1)	6.4 (3.6–9.6)
High total cholesterol	73 (51–101)	41 (21–71)	2.7 (2.0–3.7)	3.3 (2.1–4.8)	20.4 (− 10.6 to 51.1)	6.5 (4.8–8.7)	5.8 (3.8–8.4)
Impaired kidney function	52 (41–63)	34 (21–52)	1.9 (1.5–2.3)	2.7 (2.2–3.4)	42.7 (20.9 to 61.6)	4.6 (3.7–5.5)	4.8 (3.8–5.9)
**Low physical activity**	**24 (17–31)**	**17 (10–27)**	**0.9 (0.6–1.2)**	**1.4 (1.0–1.8)**	**54.1 (27.3 to 80.1)**	**2.1 (1.5–2.7)**	**2.4 (1.7–3.2)**
	**Disability-adjusted life years**						
				**All causes**	**Change in proportion (%)**	**Communicable, maternal, neonatal, and nutritional diseases**	
**Child and maternal undernutrition**	**19,040 (15,070–24,312)**	**3445 (2648–4359)**	**17.3 (13.8–22.3)**	7.4 (5.4–9.9)*	**− 57.2 (− 71.3 to − 39.9)**	**31.0 (25.0–38.4)**	17.2 (12.8–22.0)*
Childhood undernutrition	16,719 (12,967–21,821)	2667 (1971–3462)	15.2 (11.7–19.8)	5.8 (3.9–8.1)*	−62.1 (− 76.1 to − 43.9)	27.2 (21.2–34.5)	13.3 (9.3–17.9)*
Childhood wasting	13,916 (9775–19,748)	2393 (1683–3178)	12.7 (8.9–18.1)	5.2 (3.4–7.3)*	−59.2 (− 74.6 to − 36.9)	22.7 (15.6–31.8)	12.0 (8.1–16.2)
Childhood underweight	7301 (4350–12,675)	946 (594–1495)	6.6 (4.0–11.5)	2.0 (1.2–3.4)*	−69.2 (− 84.8 to − 43.8)	11.9 (7.1–20.0)	4.7 (2.9–7.7)
Childhood stunting	4700 (2097–9260)	583 (241–1157)	4.3 (1.9–8.4)	1.3 (0.5–2.5)	−70.5 (− 88.6 to − 42)	7.6 (3.5–14.5)	2.9 (1.2–5.7)
Suboptimal breastfeeding	2654 (1277–4248)	600 (308–1007)	2.4 (1.2–3.9)	1.3 (0.6–2.2)	−46.4 (− 71.6 to 6)	4.3 (2.0–7.0)	3.0 (1.5–5.0)
Non-exclusive breastfeeding	2387 (1180–3871)	562 (282–951)	2.2 (1.1–3.5)	1.2 (0.6–2.1)	−44.2 (− 70.9 to 8.6)	3.9 (1.8–6.4)	2.8 (1.4–4.8)
Discontinued breastfeeding	328 (74–771)	48 (13–110)	0.3 (0.1–0.7)	0.1 (0.0–0.3)	− 65.3 (− 85.3 to 15)	0.5 (0.1–1.3)	0.2 (0.1–0.6)
Iron deficiency	1699 (1227–2338)	665 (445–956)	1.5 (1.1–2.1)	1.4 (1.1–1.9)	−8.5 (− 27.9 to 17.8)	2.8 (2.0–3.8)	3.3 (2.5–4.4)
Vitamin A deficiency	3069 (1044–7297)	216 (78–620)	2.8 (0.9–6.7)	0.5 (0.2–1.5)	−83.2 (− 95.2 to − 32.9)	5.0 (1.7–11.5)	1.1 (0.4–3.3)
Zinc deficiency	416 (26–1200)	60 (5–161)	0.4 (0.0–1.1)	0.1 (0.0–0.4)	−65.4 (− 87.4 to 30.1)	0.7 (0.0–2.0)	0.3 (0.0–0.9)
Metabolic risks	**450 (257–709)**	**172 (81–331)**	**9.1 (8.1–10.1)**	**13.1 (10.9–15.3)**	**43.9 (16.6 to 74)**	**0.7 (0.4–1.1)**	**0.8 (0.5–1.5)**
High fasting plasma glucose	450 (257–709)	172 (81–331)	2.8 (2.5–3.3)	4.4 (3.7–5.2)	55.4 (27.4 to 84.3)	0.7 (0.4–1.1)	0.8 (0.5–1.5)
			**All causes**		**Change in proportion (%)**	**Non-communicable diseases**	
**Dietary risks**	**6678 (5308–8507)**	**3856 (2344–6154)**	**6.1 (5–7.4)**	**8.1 (6.1–10.5)**	**33.6 (3.5 to 68.3)**	**19.2 (16–23.1)**	**16.5 (12.7–21.2)**
Diet low in fruits	2563 (1729–3470)	1394 (763–2261)	2.3 (1.6–3.1)	2.9 (1.9–4.1)	25.8 (− 7.5 to 62.3)	7.4 (5.2–9.8)	6.0 (3.9–8.2)
Diet low in whole grains	1572 (997–2282)	891 (449–1579)	1.4 (0.9–2)	1.9 (1.1–2.8)	31.1 (− 3.4 to 68)	4.5 (2.9–6.4)	3.8 (2.3–5.6)
Diet low in vegetables	1520 (817–2380)	838 (365–1537)	1.4 (0.7–2.1)	1.8 (0.9–2.8)	27.3 (− 8.4to 66.9)	4.4 (2.4–6.7)	3.6 (1.8–5.7)
Diet high in sodium	1326 (247–3482)	733 (125–2106)	1.2 (0.2–3.2)	1.5 (0.3–3.9)	27.4 (− 8.9 to 68.4)	3.8 (0.7–10.0)	3.1 (0.6–8.0)
Diet low in nuts and seeds	1175 (731–1660)	736 (399–1256)	1.1 (0.7–1.5)	1.5 (0.9–2.3)	44.8 (7.9 to 86.9)	3.4 (2.2–4.7)	3.2 (2.0–4.7)
Diet low in seafood omega-3 fatty acids	879 (370–1408)	510 (184–1021)	0.8 (0.3–1.3)	1.1 (0.4–1.9)	33.7 (− 5.0 to 82.8)	2.5 (1.1–4.1)	2.2 (0.9–3.8)
Diet high in processed meat	426 (176–682)	264 (111–468)	0.4 (0.2–0.6)	0.6 (0.3–0.9)	43.5 (10.1 to 81.1)	1.2 (0.5–1.9)	1.1 (0.5–1.8)
Diet high in trans fatty acids	270 (97–530)	152 (46–346)	0.2 (0.1–0.5)	0.3 (0.1–0.6)	30.2 (− 9.8 to 77.7)	0.8 (0.3–1.5)	0.7 (0.2–1.3)
Diet low in milk	53 (18–94)	37 (12–72)	0.1 (0.0–0.1)	0.1 (0.0–0.1)	58.9 (15.7 to 114.9)	0.2 (0.1–0.3)	0.2 (0.1–0.3)
Diet suboptimal in calcium	75 (46–109)	51 (27–91)	0.1 (0.0–0.1)	0.1 (0.1–0.2)	56.9 (14.7 to 113.2)	0.2 (0.1–0.3)	0.2 (0.1–0.3)
Diet high in sugar-sweetened beverages	23 (14–35)	16 (9–28)	0.0 (0.0–0.0)	0.0 (0.0–0.1)	67.1 (25.6 to 123.7)	0.1 (0.0–0.1)	0.1 (0.1–0.1)
Diet low in polyunsaturated fatty acids	19 (8–31)	11 (4–21)	0.0 (0.0–0.0)	0.0 (0.0–0.0)	36.0 (− 1.3 to 81.2)	0.1 (0.0–0.1)	0.1 (0.0–0.1)
Diet low in fiber	7 (1–20)	4 (0–13)	0.0 (0.0–0.0)	0.0 (0.0–0.0)	35.9 (− 22.1 to 128.7)	0.0 (0.0–0.1)	0.0 (0.0–0.1)
**Metabolic risks**	**9172 (7725–10,701)**	**5804 (3840–8452)**	**9.1 (8.1–10.1)**	13.1 (10.9–15.3)*	**43.9 (16.6 to 74.0)**	**26.4 (24.0–28.8)**	**24.9 (21.2–28.7)**
High systolic blood pressure	5715 (4690–6830)	3582 (2286–5400)	5.2 (4.4–6.0)	7.5 (5.9–9.3)	45.1 (13.2 to 80.5)	16.4 (14.2–18.7)	15.4 (12.3–18.5)
High fasting plasma glucose	2680 (2224–3207)	1921 (1310–2753)	2.8 (2.5–3.3)	4.4 (3.7–5.2)*	55.4 (27.4 to 84.3)	7.7 (6.8–8.8)	8.3 (7.1–9.7)
High body-mass index	910 (383–1584)	1209 (601–2124)	0.8 (0.4–1.5)	2.5 (1.4–3.8)	207.9 (111.5 to 387.3)	2.6 (1.2–4.5)	5.2 (3.0–7.7)
High total cholesterol	1780 (1305–2387)	826 (442–1374)	1.6 (1.2–2.1)	1.7 (1.1–2.5)	7.2 (− 25.9 to 44.4)	5.1 (3.9–6.5)	3.5 (2.3–5.1)
Impaired kidney function	1143 (937–1388)	703 (471–1025)	1.0 (0.9–1.2)	1.5 (1.2–1.8)	43.0 (17.4 to 68.1)	3.3 (2.8–3.8)	3.0 (2.5–3.6)
**Low physical activity**	**502 (351–672)**	**339 (210–531)**	**0.5 (0.3–0.6)**	**0.7 (0.5–1.0)**	**56.9 (22.5 to 95.7)**	**1.4 (1.0–1.9)**	**1.5 (1.0–1.9)**

*UI* uncertainty interval, *-shows a significant change; (i.e. changes were based on 95% UI–out of the UI); Zero (0.0) indicates very low proportion. The sum of percentages of risk factors in a column exceeds the total for all risk factors combined under a risk factor cluster because of an overlap between various risk factors

Over the 25 years, the proportion of all-cause and CMNND DALYs attributable to CMU decreased from 17.3% [13.8–22.3] to 7.4% [5.4–9.9] and from 31.0% [25.0–38.4] to 17.0% [12.8–22.0], respectively. However, the proportion of all-cause and NCD DALYs attributable to DRs and MRs increased from 6.1% [5.0–7.4] to 8.1% [6.1–10.5] and from 9.1% [8.1–10.1] to 13.1 [10.9–15.3], respectively (Table 3 and Additional file 1: Figures S2 and S3).

### Comparison with other East African countries

Comparison of the disease burden attributable to CMU, DRs, MRs and LPA among each of the 15 East African countries is shown in Table 4. In all countries, the age-standardized proportion of mortality attributable to CMU decreased, with the highest reduction (74.0%) in Mozambique. In 2015, Ethiopia ranked ninth and 12^th^ in terms of age-adjusted PAF of deaths and DALYs attributable to CMU, respectively. Increases in the burden of disease associated with DRs and MRs were recorded for almost all countries. In 2015, Ethiopia was among the top four Eastern SSA countries with the highest burden of disease (both in terms of deaths and DALYs) based on the age-standardized proportion of disease attributable to DRs, MRs and LPA. Between 1990 and 2015, the country showed a 35.0%, 42.5% and 54.1% increase in age-standardized proportion of deaths attributable to DRs, MRs and LPA, respectively. In 2015, the age-specific pattern of disease burden (PAF and rates of death and DALYs) attributable to MRs and LPA in Ethiopia was similar with the average age-specific estimates pattern of Eastern SSA countries. The pattern of age-specific disease burden attributable to DRs was relatively higher for Ethiopia compared to the average of Eastern SSA countries, although it was not significantly different based on the uncertainty intervals (data not shown).

**Table 4:** Age-standardized proportion (95% uncertainty interval) of all deaths and disability-adjusted life years attributable to child and maternal undernutrition, low physical activity, dietary and metabolic risk factors and rank of East African countries between 1990 and 2015

Risk factors		Deaths					Disability-adjusted life years (DALYs)				
		1990		2015			1990		2015		
		Proportion (%) of all deaths (95% UI)	Rank	Proportion (%) of all deaths (95% UI)	Rank	Change (%) (95% UI)	Proportion (%) of all DALYs (95% UI)	Rank	Proportion (%) of all DALYs (95% UI)	Rank	Change (%) (95% UI)
Child and maternal undernutrition	Somalia	11.8 (7.1–19.4)	2	7.5 (4.7–12.6)	1	−36.6 (− 68.1 to 20.5)	21.8 (14.2–30.1)	2	15.5 (9.6–22.7)	1	−28.8 (−58.5 to 19.3)
	South Sudan	13.7 (7.5–22.2)	1	6.9 (3.9–11.3)	2	−49.3 (−75.0 to 2.7)	23.6 (15.1–32.8)	1	13.3 (7.7–19.2)	2	−43.4 (−66.6 to −5.8)
	Eritrea	9.9 (8.3–12.0)	4	5.0 (3.6–7.2)*	3	−49.2 (−64.1 to −24)	20.5 (17.5–24.4)	3	10.9 (7.7–14.8)*	3	−46.9 (−62.7 to − 25.3)
	Djibouti	9.3 (7.0–12.2)	5	4.9 (3.1–7.2)	4	−47.5 (−68.8 to −14.3)	18.3 (14.6–22.4)	5	10.6 (7.1–14.4)*	4	−42.1 (−62.6 to −14.7)
	Madagascar	10.2 (8.8–12.4)	3	4.7 (3.3–6.6)*	5	−54.1 (−68 to −35)	20.1 (17.5–23.9)	4	10.4 (7.7–13.8)*	5	−48.2 (−62.6 to − 30.1)
	Burundi	5.8 (4.4–7.6)	14	4.7 (3.2–6.9)	6	−19.3 (−49.6 to 25.3)	11.2 (8.4–14.5)	14	9.2 (6.5–13.0)	6	−17.9 (−46.7 to 25.1)
	Malawi	9.2 (6.9–11.8)	6	4.4 (3.2–6.0)*	7	−52.3 (−67.6 to − 28.1)	15.9 (12.0–20.3)	8	8.4 (6.4–10.8)*	7	−47.5 (− 63.2 to − 23.9)
	Rwanda	6.9 (5.2–8.6)	12	4.2 (2.9–5.9)	8	−39.2 (−59.2 to −8.9)	13.5 (10.2–17.1)	12	8.3 (6.2–11.1)	8	−38.6 (− 57.5 to − 10.7)
	**Ethiopia**	**8.6 (6.8–11.0)**	**8**	3.6 (2.5–5.1)*	**9**	**−58.5 (−72.4 to − 39.7)**	**17.3 (13.8–22.3)**	**6**	7.4 (5.4–9.9)*	**12**	**− 57.2 (− 71.3 to − 39.9)**
	Tanzania	8.7 (7.1–10.6)	7	3.5 (2.6–4.8)*	10	− 59.6 (−71.3to − 42.9)	17.1 (14.2–20.2)	7	7.6 (5.8–9.9)*	9	− 55.4 (−68 to − 40.3)
	Kenya	6.4 (5.7–7.3)	13	3.5 (2.9–4.1)*	11	−45.8 (− 53.1 to − 37.9)	13.5 (12.2–15)	13	7.5 (6.6–8.6)*	10	−44.3 (− 50.5 to − 37.8)
	Comoros	7.9 (5.9–11.1)	10	3.4 (2.4–4.8)*	12	−56.7 (− 72.4 to − 31.1)	15.8 (12.2–20.4)	9	7.5 (5.4–10.1)*	11	− 52.6 (− 67.9 to − 27.8)
	Uganda	4.8 (3.7–6.2)	15	2.9 (2.1–4.2)	13	− 39.7 (− 60.3 to − 8.5)	9.4 (7.3–11.6)	15	6.1 (4.5–8.2)	13	−35.2 (− 56.0 to − 6.4)
	Zambia	8.0 (6.4–9.8)	9	2.8 (2.1–3.6)*	14	− 65.4 (− 74.7 to − 52.8)	14.8 (11.8–17.8)	10	5.2 (4.0–6.7)*	14	−64.7 (− 74.2 to − 51.5)
	Mozambique	7.8 (6.1–10.1)	11	2.0 (1.5–2.8)*	15	−74.0 (− 82.2 to −62.0)	14.1 (11.2–17.9)	11	4.5 (3.4–5.8)*	15	−68.1 (− 77.6 to −55.3)
	Eastern Sub-Saharan Africa	8.1 (7.1–9.2)		3.7 (3.2–4.3)*		−54.1 (−61.3 to − 45.6)	15.8 (13.9–17.9)		7.8 (6.9–8.9)*		−50.8 (− 58.3 to − 42.7)
Dietary risks	Madagascar	14.2 (11.6–18.1)	2	16.1 (12.6–20.7)	1	13.2 (0.0 to 24.6)	7.7 (6.3–9.5)	1	9.7 (6.9–13.0)	1	25.9 (− 3.4 to 56.5)
	Tanzania	13.7 (11.0–16.6)	3	15.9 (12.7–19.3)	2	16.4 (2.3 to 31.6)	6.6 (5.4–8.0)	6	8.4 (6.1–11.0)	3	27.1 (−4.1 to 65.6)
	Djibouti	14.4 (11.8–17.8)	1	15.0 (12.2–18.5)	3	4.0 (−10 to 22.2)	7.5 (5.6–9.8)	3	8.6 (6.0–11.6)	2	13.8 (−21.6 to 67.8)
	**Ethiopia**	**10.8 (8.8–13.3)**	**7**	**14.5 (11.7–18.0)**	**4**	**35.0 (14.1 to 53.9)**	**6.1 (5.0–7.4)**	**7**	**8.1 (6.1–10.5)**	**4**	**33.6 (3.5 to 68.3)**
	Comoros	13.5 (10.9–17.0)	4	13.4 (10.6–17.3)	5	−0.7 (− 13.6 to 13.8)	7.7 (5.7–10.2)	2	7.9 (6.0–10.2)	5	2.6 (− 23.7 to 37.6)
	Eritrea	11.4 (9.1–14.5)	6	12.8 (10.3–16.2)	6	12.5 (−0.3 to 25.3)	6.6 (5.4–8.4)	5	7.8 (5.6–10.2)	6	17.5 (−13.6 to 45.2)
	Zambia	9.1 (7.2–11.5)	11	12.7 (10.5–15.5)	7	39.4 (19.3 to 62.8)	4.5 (3.5–5.7)	11	7.7 (5.9–9.7)*	7	71.5 (31.4 to 117.5)
	Burundi	11.8 (9.1–15.4)	5	11.5 (8.7–14.8)	8	−2.9 (− 23.6 to 16.9)	7.4 (5.4–9.9)	4	6.8 (4.9–9.1)	8	−7.9 (−33.9 to 27.1)
	Mozambique	8.9 (6.6–11.5)	12	11.4 (8.6–14.7)	9	28.5 (3.6 to 56.6)	4.1 (3.1–5.3)	12	6.3 (4.2–8.7)	9	52.5 (6.0 to 103.2)
	Somalia	10.5 (7.2–13.8)	8	10.6 (7.9–13.3)	10	0.6 (−20.7 to 33.3)	5.7 (2.9–8.5)	8	6.2 (3.5–8.9)	10	9.1 (−37.5 to 113.7)
	Rwanda	9.6 (7.2–13.2)	9	10.2 (7.2–13.9)	11	5.3 (−12.9 to 23.1)	5.5 (4.1–7.4)	9	5.4 (3.7–7.7)	11	−0.5 (−25.6 to 33.3)
	South Sudan	9.6 (6.8–12.9)	10	9.9 (7.2–13.2)	12	2.3 (−22.7 to 36.2)	4.8 (2.6–7.5)	10	5.4 (3.3–8.4)	12	14.5 (−38.3 to 120.3)
	Uganda	6.7 (4.8–9.0)	14	9.0 (6.8–11.8)	13	35.5 (13.0 to 60.1)	3.4 (2.3–4.7)	13	5.2 (3.4–7.0)	13	51.7 (9.5 to 101.4)
	Malawi	6.9 (5.5–8.4)	13	8.3 (6.3–10.2)	14	19.8 (−0.6 to 43.3)	3.3 (2.5–4.0)	15	4.4 (3.1–5.9)	14	35.2 (0.6 to 82.5)
	Kenya	6.0 (5.1–7.2)	15	6.7 (5.6–7.9)	15	10.5 (3.9 to 18.4)	3.4 (2.8–4.0)	14	3.9 (3.2–4.6)	15	15.1 (4.6 to 26.7)
	Eastern Sub-Saharan Africa	10.2 (8.5–12.5)		12.4 (10.2–15.1)		21.2 (10.8 to 31.2)	5.5 (4.6–6.6)		6.9 (5.7–8.5)		26.8 (10.2 to 43.4)
Metabolic risks	Madagascar	24 (22.2–25.7)	2	28.3 (25.6–30.6)	1	17.9 (8.2 to 26.9)	12.3 (11.2–13.4)	2	16.1 (12.9–19.0)	1	31.2 (5.5 to 56.9)
	Djibouti	23.7 (21.5–26.1)	3	26.9 (24.5–29.5)	2	13.8 (2.0 to 27.5)	12.0 (9.7–14.6)	3	15.0 (11.8–18.5)	2	25.2 (−7.1 to 68.5)
	Comoros	24.4 (22.1–26.7)	1	25.8 (23.3–28.1)	3	5.4 (−5.9 to 18.0)	13.3 (10.5–15.8)	1	14.7 (12.5–17.1)	3	10.8 (−12.7 to 40.9)
	**Ethiopia**	**17.0 (15.4–18.7)**	**12**	24.2 (22.2–26.1)*	**4**	**42.5 (27.1 to 58.3)**	**9.1 (8.1–10.1)**	**8**	13.1 (10.9–15.3)*	**4**	**43.9 (16.6 to 74)**
	Rwanda	20.4 (17.9–23.2)	4	22.3 (19.9–25.1)	5	9.4 (−5.0 to 25.1)	10.8 (9.2–12.4)	5	11.5 (9.5–13.7)	10	6.6 (−15.4 to 34.2)
	Tanzania	18.2 (16.6–19.8)	8	22.2 (19.6–24.8)	6	21.9 (7.2 to 37.2)	8.8 (7.8–9.7)	9	11.9 (9.5–14.7)	8	35.5 (6.8 to 69.6)
	Mozambique	17.5 (15.7–19.2)	10	22 (18.7–25.0)	7	26.1 (5.8 to 47.1)	7.8 (7.0–8.8)	12	11.8 (8.8–14.6)*	9	50.0 (10.8 to 89.7)
	Eritrea	19.0 (17.1–21.1)	6	21.7 (19.3–24.2)	8	14.1 (1.8 to 27.9)	10.5 (9.3–11.8)	6	12.6 (9.8–14.9)	6	20.1 (−6.8 to 42.3)
	Zambia	16.2 (14.3–18.3)	13	21.7 (19.5–23.6)*	9	33.3 (16.6 to 52.9)	7.7 (6.5–8.9)	13	12.7 (10.5–14.5)*	5	64.3 (32.1 to 101.2)
	Burundi	20.3 (17.6–24.0)	5	21.4 (19.0–23.9)	10	5.2 (−10.9 to 22.4)	11.8 (9.6–14.5)	4	11.9 (9.7–14.1)	7	0.9 (−22.2 to 31.4)
	Uganda	17.0 (14.5–19.1)	11	21.2 (18.5–23.7)	11	24.2 (8.2 to 43.7)	8.1 (6.3–9.5)	11	11.4 (8.7–13.8)	11	40.4 (8.0 to 77.7)
	Somalia	18.4 (14.1–21.3)	7	19.2 (16.1–21.7)	12	4.7 (−12.8 to 32.8)	9.5 (5.5–12.8)	7	10.8 (7.2–13.7)	12	14.6 (−27.8 to 103.4)
	Malawi	15.8 (13.9–17.7)	14	19 (16.3–21.4)	13	20.0 (2.3 to 41.3)	7.0 (5.9–8.2)	15	9.5 (7.5–11.8)	14	35.3 (4.6 to 73.3)
	South Sudan	17.7 (13.8–20.7)	9	18.6 (15.5–21.3)	14	5.5 (−15.0 to 35.5)	8.4 (5.2–12.0)	10	10.0 (6.9–13.6)	13	18.9 (−28.6 to 108.2)
	Kenya	14.7 (13.5–16.0)	15	16.7 (15.6–17.9)	15	14.1 (7.8 to 21.2)	7.6 (7.0–8.4)	14	9.2 (8.3–9.9)	15	19.8 (11.0 to 29.2)
	Eastern Sub-Saharan Africa	17.7 (16.4–19.0)		22.1 (20.8–23.4)*		24.7 (17.6 to 33.1)	9.0 (8.4–9.8)		12.0 (10.9–13.2)*		33.0 (20.3 to 46.2)
Low physical activity	Djibouti	1.3 (0.9–1.7)	1	1.8 (1.3–2.3)	1	35.9 (14.9 to 63.2)	0.6 (0.4–0.9)	1	0.9 (0.7–1.3)	2	48.6 (8.3 to 104.0)
	Zambia	1.1 (0.8–1.4)	3	1.7 (1.3–2.1)	2	53.7 (33.4 to 78.3)	0.5 (0.4–0.7)	3	1.0 (0.7–1.3)*	1	89.5 (52.0 to 132.4)
	**Ethiopia**	**0.9 (0.6–1.2)**	**7**	**1.4 (1.0–1.8)**	**3**	**54.1 (27.3 to 80.1)**	**0.5 (0.3–0.6)**	**7**	**0.7 (0.5–1.0)**	**4**	**56.9 (22.5 to 95.7)**
	Madagascar	1.1 (0.8–1.5)	2	1.3 (0.9–1.8)	4	20.4 (5.1 to 35.6)	0.5 (0.4–0.7)	2	0.7 (0.5–1.0)	3	36.7 (9.4 to 65.4)
	Rwanda	1.0 (0.6–1.3)	4	1.2 (0.8–1.6)	5	24.3 (4.5 to 46.7)	0.5 (0.3–0.7)	6	0.6 (0.4–0.8)	7	22.5 (−3.2 to 55.5)
	Comoros	0.9 (0.6–1.3)	5	1.1 (0.8–1.5)	6	20.4 (4.2 to 40.1)	0.5 (0.3–0.7)	5	0.6 (0.4–0.8)	5	28.9 (1.3 to 62.8)
	Tanzania	0.9 (0.6–1.1)	11	1.1 (0.8–1.5)	7	34.3 (17.8 to 55.0)	0.4 (0.3–0.5)	11	0.6 (0.4–0.8)	8	48.0 (17.0 to 87.1)
	Mozambique	0.8 (0.5–1.1)	12	1.1 (0.8–1.4)	8	41.9 (16.5 to 72.6)	0.3 (0.2–0.5)	13	0.6 (0.4–0.8)	10	69.3 (26.4 to 120.2)
	Eritrea	0.9 (0.6–1.2)	10	1.1 (0.7–1.4)	9	24.3 (6.7 to 43.2)	0.5 (0.3–0.6)	8	0.6 (0.4–0.8)	6	34.0 (6.4 to 62.6)
	Uganda	0.7 (0.5–1.0)	14	1.1 (0.8–1.4)	10	47.6 (25.2 to 77.0)	0.3 (0.2–0.5)	14	0.6 (0.4–0.8)	11	68.6 (29.7 to 116.2)
	Burundi	0.9 (0.6–1.2)	6	1.0 (0.7–1.4)	11	15.0 (−9.2 to 37.7)	0.5 (0.3–0.7)	4	0.6 (0.4–0.8)	9	14.5 (−12.9 to 49.2)
	Malawi	0.8 (0.5–1.0)	13	1.0 (0.7–1.4)	12	31.9 (11.8 to 57.8)	0.3 (0.2–0.5)	12	0.5 (0.3–0.7)	13	50.6 (18.1 to 94.7)
	South Sudan	0.9 (0.6–1.2)	8	1.0 (0.7–1.3)	13	16.1 (−4.8 to 45.8)	0.4 (0.2–0.6)	10	0.5 (0.3–0.8)	12	32.7 (−17.5 to 117.6)
	Somalia	0.9 (0.6–1.2)	9	0.9 (0.7–1.3)	14	6.3 (−11.5 to 30.6)	0.4 (0.2–0.6)	9	0.5 (0.3–0.7)	14	18.4 (−21.5 to 96.6)
	Kenya	0.6 (0.4–0.8)	15	0.7 (0.5–0.9)	15	16.8 (8.4 to 26.6)	0.3 (0.2–0.4)	15	0.4 (0.3–0.5)	15	22.3 (11.6 to 33.7)
	Eastern Sub-Saharan Africa	0.9 (0.6–1.1)		1.2 (0.8–1.5)		36.2 (24.4 to 48.6)	0.4 (0.3–0.5)		0.6 (0.4–0.8)		45.9 (29.8 to 63.7)

*DALYs* Disability-adjusted life years, *UI* uncertainty interval; * -shows a significant change; (i.e. changes were based on 95% UI–out of the UI)

## Discussion

This systematic investigation of disease burden attributable to CMU, DRs, MRs and LPA in Ethiopia and the comparison with other 14 Eastern SSA countries provides, for the first time, a comprehensive picture of mortality and DALYs attributable to nutritional, lifestyle and metabolic factors. Despite a significant reduction in CMU-attributable disease, there was an increasing trend for the burden of disease attributable to DRs, LPA, and MRs in the country. Our findings show that while a high burden of CMU-attributable disease still remains, the burden attributable to DRs, LPA and MRs is also high and continues to increase. We found that 9%, 10%, and 15% of all-cause mortality in Ethiopia were attributable to CMU, DRs, and MRs, respectively. Ethiopia was among four Eastern SSA countries with the highest burden of disease (both in terms of deaths and DALYs) based on the age-standardized proportion of disease attributable to DRs, MRs and LPA in 2015. These results call for an increased investment in prevention and control of the aforementioned risk factors.

Of the 17 risk factors (level 2) in GBD 2015 [38], DRs, high SBP and CMU were the second (behind air pollution), third and fourth most common risk factors of deaths in Ethiopia, respectively. In terms of DALYs, CMU was still a leading risk factor in the country. DRs and high SBP were the fifth (behind CMU, air pollution, unsafe water, sanitation, and handwashing, and unsafe sex) and sixth most common risk factors, respectively. High FPG (seventh), overweight/obesity (ninth), high total cholesterol (11^th^) and low GFR (12^th^) were also common risk factors of deaths in the country. However, of the seven clusters (level 2) of behavioral risk factors, DRs were the leading contributors for deaths in the country and the third (behind CMU and unsafe sex) for DALYs [38].

In our study, we found that, based on age-standardized proportion of death in 2015, DRs and MRs were responsible for more deaths than the CMU in Ethiopia. Diets low in fruits, vegetables, nuts and seeds, and whole grains and high in sodium were the most common five DRs, each accounting for 2% or more of all-cause deaths, and more than 4% of all-cause DALYs in the country. It is worth noting that the issue of diet-related NCD burden is not only a concern among high-income countries [2, 39]. In our previous study, we have discussed the need for coordinated efforts to reduce the high burden of disease related to DR factors of NCDs in Ethiopia [37]. Similarly, a recent (2016) report [40] indicated that the consumption of fruit and vegetables was very minimal—only 2.4% of the population aged 15–65 years old consumed five or more servings of fruit and vegetables per day in Ethiopia. In 2011, it is reported that cereals (“Teff”, wheat, barley, maize, sorghum and other cereals) contributed to the majority (43%) of food consumed (kg/adult equivalent/year) in Ethiopia, while the consumption of vegetables and fruits (10.5%), pulses (5.1%), animal products (4.6%) and oilseeds (0.1%) was low [41]. This suggests that the food pattern in the country is “monotonous” and minimally diversified, which increases the risk of susceptibility for NCDs [42], micronutrient deficiencies and their associated adverse health outcomes [43].

In addition, the prevalence of alcohol consumption (consumption of alcohol in the past 30 days), tobacco smoking [current tobacco users (daily and non-daily)], and LPA (less than 600 metabolic equivalent time-minutes) in Ethiopia was 41%, 4%, and 6%, respectively [40]. MRs are also major contributors to the high burden of NCDs in the country. A considerable prevalence of overweight/obesity (16%), high triglyceride [> 150 mg/dl] (21%), raised blood pressure (22%) and high FPG or diabetes (6%) has been reported [40], which suggests the public health importance of these risk factors. The GBD study also found that the prevalence of overweight/obesity among people 20 years and above has significantly increased from 6% (5–7) in 1990 to 14% (12–17) in 2015 in Ethiopia [44]. This highlights the need for coordinated effort and investment to improve dietary quality, create a favorable environment for physical activity and implement strategies for early screening and control of MRs in the country parallel to the continuing investments on CMU.

Although the burden of CMU-attributable disease has decreased over the past 25 years, the burden is still significantly high in Ethiopia. At the same time, there is also a high burden of NCDs attributable to suboptimal diet and MR factors in the country. Whereas the age-adjusted proportion of mortality due to NCDs in Ethiopia significantly increased by 35%, from 42% (39–45) in 1990 to 57% (54–60) in 2015, deaths due to CMNNDs decreased by 23%, from 47% (44–50) to 36% (33–39) over the same time frame [38]. In addition to the increased burden of DR, lifestyle and MR factors, multiple determinants could have contributed to the dynamics of disease burden in the country. Health and health-related interventions (including policies and related investments) in curbing and controlling CMNNDs and the risk factors in the country might have contributed to the current pattern of disease burden [27, 45]. The increased life expectancy [6] in the country could be a proxy evidence for the changes in disease dynamics and the impact of the interventions. Between 1990 and 2015, the estimated life-expectancy at birth increased from 43 to 64 years [46], and it was the top-ranked increase.

Furthermore, previous exposure to food deprivation and undernutrition (particularly during childhood) could exacerbate the current burden of lifestyle and metabolic diseases. It is well recognized that childhood undernutrition increases the susceptibility of adults to NCDs (“fetal origin” hypothesis) [47–49]. Several mechanisms were proposed for this association, including structural changes of organs [50], preference for fatty foods and increased risk of dyslipidemia [51], and epigenetic change [13, 14]. Epidemiological studies have demonstrated that early famine exposure increased the risk for a range of NCDs [52–55], metabolic diseases [52, 54, 56, 57] and overweight/obesity [57]. Evidence also shows that an early-life exposure to food deprivation could modify the risk of complications of NCDs [58]. Evidence of the impact of previous famine history on the current burden of disease in Ethiopia is lacking, warranting further investigations in the area.

Ethiopia has successfully implemented large-scale measures that have improved public health, particularly in reducing CMNNDs [2]. For instance, the country achieved MDG-5 (reducing under-five mortality by two-thirds) in 2013 [25]. Successful public health strategies on the risk factors of child mortality [2, 25, 31] through expansion of primary health care has brought a significant reduction in child mortality. Our study also highlighted that CMU-attributed mortality and DALYs have significantly declined over the past 25 years, although the burden is still significantly high. At the same time, diet-, lifestyle- and overweight/obesity-related diseases are high and increasing, creating a further challenge in the health care system and services of the country. Compared to other Eastern SSA countries, Ethiopia was among the top four countries with high burden of disease attributable to DRs, MRs and LPA, while Kenya had the lowest burden of disease related to these risk factors. In addition to its national health policy, the Kenyan government and its partners have developed a national strategy for prevention and control of NCDs (2015–2020) [59] with the purpose of providing more specific emphasis on interventions against NCDs and their risk factors. This strategic plan provides a platform to specifically focus on reducing NCD risk factors, allocating resources and designing tailored and coordinated interventions against NCDs.

In Ethiopia, although there have been efforts to prevent and control NCDs [27, 60], interventions against the high and growing burden of disease attributable to DRs, lifestyle, and MRs is minimal. Therefore, while continuing to invest in CMU-attributed disease and the risk factors, it is imperative to design appropriate response for the growing burden of disease related to suboptimal diet, lifestyle and MR factors in line with the global initiatives [17, 23, 24]. In addition to the Health Sector Transformation Plan [27], designing a comprehensive and evidence based plan that specifically targets reducing NCD burden and risk factors by sharing experience from other countries with a similar environment (such as Kenya) is necessary. Revision of the NCD strategic framework [30] based on latest evidence should be undertaken. Dietary guidelines and policy should be developed to promote and guide healthy eating behaviors and regulate food supply in the country. Further, despite the high burden of disease attributable to suboptimal diet and MR, data on food and nutrient intake, behavioral factors and metabolic markers of NCDs in Ethiopia are very limited. As part of the investments, the gap in nationally representative individual level data on these key risk factors should be addressed by establishing a national level surveillance system and expanding (in terms of both coverage and depth) health and demographic surveillance sites in the country. Studies should also further investigate the impact of previous food deprivation and undernutrition on the current burden of NCDs in the country.

This study is not without limitations in spite of the fact that we have used data from GBD that uses robust methods to collate and analyze data. Detailed methodological shortfalls of the overall estimation process and risk factors have been discussed elsewhere [2, 37]. Specific limitations to the current study are discussed here. First, shortage of data, particularly on DRs, MRs and LPA, is a major challenge to estimating the exposure level. To assist in addressing this, the modeling strategies ST-GPR and DisMod-MR 2.1 were used, where data from the region, super-region and global levels contributed to country level estimates. However, the uncertainty intervals of the estimates are wider for these risk factors. Secondly, we did not assess the difference between urban versus rural area estimates, and differences across regions, as the distribution of risk factors and disease burden could potentially be different in these settings [40]. Thirdly, the correlation among risk factors (for instance, among DR factors) is another potential limitation. Fourthly, residual confounding could affect the estimates of relative risks. Lastly, the use of similar effect size (relative risks) across countries (for a given age-sex category) could be a potential drawback as the effect of a risk factor in different population groups could have a different effect on a disease outcome [2].

## Conclusions

In summary, while Ethiopia has significantly reduced the burden of CMU attributable disease, the burden attributable to DRs and MRs of NCDs, and LPA increased over the past 25 years. However, despite the reduction, the burden of disease attributable to CMU is still a public health problem. In 2015, a higher age-standardized proportion of deaths were due to diseases attributable to DRs and MRs of NCDs compared to those CMU-attributed. This reflects the country’s success and challenge in curbing nutrition-, metabolic- and lifestyle-related diseases. To effectively mitigate the challenge, policies should target DRs, MRs and other behavior-related factors in addition to the efforts to reduce undernutrition. Given the enormous cost of diseases associated with lifestyle factors and MRs for the individual and the healthcare system, future investments on the scaling-up of prevention and control of these risk factors are required to effectively address the growing health challenge in Ethiopia. Policies and guidelines should be designed to mitigate the behavioral and MR factors during the SDG era. A collective and integrated multi-sectoral approach is required to successfully prevent and control NCDs and their risk factors. Lifestyle and MRs of NCDs also require more attention in the primary health care system. In this regard, replicating the primary health care approach, which has been successful in reducing CMNND, could bring further success in prevention and control of NCDs and their risk factors. The establishment of a community-based national surveillance system to collate determinants of NCDs in the country, such as dietary, behavioral and metabolic factors, will further assist the anticipated interventions.

## Additional file

Additional file 1: **Table S1.** Risk factors, definitions, minimum theoretical risk exposure levels and data representative index. **Table S2.** Data sources used for estimation of mortality and disability-adjusted life years associated with maternal and child under nutrition, low physical activity, dietary and metabolic risk factors in Ethiopia in the Global Burden of Disease (GBD) Study. **Table S3.** Covariates and mediators used in Global Burden of Disease 2015 dietary risk factors study. **Figure S1.** Rate and proportion of deaths and disability-adjusted life years (DALYs) attributable to high body mass index by age in Ethiopia, 2015 (*Proportion was calculated out of all-cause of death*). **Figure S2.** Trend of mortality and DALYs (age-standardized rate and proportion) attributable to child and maternal undernutrition (CMU), dietary and metabolic risks in Ethiopia, 1990–2015 (*Proportion was calculated out of all-cause of death*). **Figure S3.** Trend of mortality and DALYs (age-standardized rate and proportion) attributable to low physical activity in Ethiopia, 1990–2015 (*Proportion was calculated out of all-cause of death*). (PDF 962 kb)

## Abbreviations

BMI: Body mass index
CMNND: Communicable, maternal, neonatal, nutritional diseases
CMU: Child and maternal undernutrition
DALYs: Disability-adjusted life years
DR: Dietary risk
FAO: Food and Agriculture Organization
FPG: Fasting plasma glucose
GBD: Global Burden of Disease
LPA: Low physical activity
MR: Metabolic risk
NCD: Noncommunicable disease
SBP: Systolic blood pressure
SSA: Sub-Saharan Africa
TMREL: Theoretical minimum-risk exposure levels
WHO: World Health Organization
