# Supplementary material for: Burden of disease attributable to suboptimal diet, metabolic risks and low physical activity in Ethiopia and comparison with Eastern sub-Saharan African countries, 1990–2015: findings from the Global Burden of Disease Study 2015
Source: BMC Public Health. 2018 Apr 25;18:552. doi: 10.1186/s12889-018-5438-1 (PMC5922000; doi:10.1186/s12889-018-5438-1)
Supplement: Supplementary file 1 — Table S1. Risk factors, definitions, minimum theoretical risk exposure levels and data representative index. Table S2. Data sources used for estimation of mortality and disability-adjusted life years associated with maternal and child under nutrition, low physical activity, dietary and metabolic risk factors in Ethiopia in the Global Burden of Disease (GBD) Study. Table S3. Covariates and mediators used in Global Burden of Disease 2015 dietary risk factors study. Figure S1. Rate and proportion of deaths and disability-adjusted life years (DALYs) attributable to high body mass index by age in Ethiopia, 2015 (Proportion was calculated out of all-cause of death). Figure S2. Trend of mortality and DALYs (age-standardized rate and proportion) attributable to child and maternal undernutrition (CMU), dietary and metabolic risks in Ethiopia, 1990–2015 (Proportion was calculated out of all-cause of death). Figure S3. Trend of mortality and DALYs (age-standardized rate and proportion) attributable to low physical activity in Ethiopia, 1990–2015 (Proportion was calculated out of all-cause of death). (PDF 962 kb) [file 12889_2018_5438_MOESM1_ESM.pdf]

Table S1: Risk factors, definitions, minimum theoretical risk exposure levels and data representative index (**source:** *GBD 2015 Risk Factors Collaborators. Global, regional, and national comparative risk assessment of 79 behavioural, environmental and occupational, and metabolic risks or clusters of risks, 1990–2015: a systematic analysis for the Global Burden of Disease Study 2015. The Lancet. 2016 Oct 7; 388:1659–1724.*)

**Risk factors**	**Definition**	**Theoretical minimum risk exposure level**	**Data representative index**
**Child and maternal undernutrition**			93.9%
**Suboptimal breastfeeding**			77·8%
Non-exclusive breastfeeding	Proportion of children younger than 6 months who receive predominant, partial, or no breastfeeding	All children are exclusively breastfed for first 6 months of life	77·8%
Discontinued breastfeeding	Proportion of children aged 6–23 months who do not receive any breastmilk	All children continue to receive breastmilk until 2 years of age	79·2%
**Childhood undernutrition**			79·3%
Childhood underweight	Proportion of children less than –3 SDs, –3 to –2 SDs, and –2 to –1 SDs of the WHO 2006 standard weight-for-age curve	All children are above –1 SD of the WHO 2006 standard weight-for-age curve	78·8%
Childhood wasting	Proportion of children less than –3 SDs, –3 to –2 SDs, and –2 to –1 SDs of the WHO 2006 standard weight-for-length curve	All children are above –1 SD of the WHO 2006 standard weight-for-height curve	79·3%
Childhood stunting	Proportion of children less than –3 SDs, –3 to –2 SDs, and –2 to –1 SDs of the WHO 2006 standard height-for-age curve	All children are above –1 SD of the WHO 2006 standard height-for-height curve	93·7%
Iron deficiency	Peripheral blood haemoglobin concentration in g/L	Country specific	68·3%
Vitamin A deficiency	Proportion of children aged 28 days to 5 years with serum retinol concentration <0.7 μmol/L	No childhood vitamin A deficiency	40·9%
Zinc deficiency	Proportion of the population with inadequate zinc intake versus loss	No inadequate zinc intake	84·3%
**Overall dietary risks**			92·9%
Diet low in fruits	Average daily consumption of fruits (fresh, frozen, cooked, canned, or dried, excluding fruit juices and salted or pickled fruits)	Consumption of fruit between 200 g and 300 g per day	88·9%
Diet low in vegetables	Average daily consumption of vegetables (fresh, frozen, cooked, canned, or dried vegetables, including legumes but excluding salted or pickled vegetables, juices, nuts and seeds, and starchy vegetables such as potatoes or corn)	Consumption of vegetables between 340 g and 500 g per day	88·9%
Diet low in whole grains	Average daily consumption of whole grains (bran, germ, and endosperm in their natural proportion) from breakfast cereals, bread, rice, pasta, biscuits, muffins, tortillas, pancakes, and other sources	Consumption of whole grains between 100 and 150 g per day	16·2%
Diet low in nuts and seeds	Average daily consumption of nut and seed foods	Consumption of nuts and seeds between 16 g and 25 g per day	88·9%
Diet low in milk	Average daily consumption of milk, including non-fat, low fat, and full-fat milk, excluding soy milk and other plant derivatives	Consumption of milk between 350 g and 520 g per day	88·9%
Diet high in red meat	Average daily consumption of red meat (beef, pork, lamb, and, goat but excluding poultry, fish, eggs, and all processed meats)	Consumption of red meat between 18 g and 27 g per day	88·9%
Diet high in processed meat	Average daily consumption of meat preserved by smoking, curing, salting, or addition of chemical preservatives	Consumption of processed meat between 0 g and 4 g per day	27·3%

Diet high in sugar sweetened beverages	Average daily consumption of beverages with ≥50 kcal per 226.8 g serving, including carbonated beverages, sodas, energy drinks, and fruit drinks, but excluding 100% fruit and vegetable juices	Consumption of sugar-sweetened beverages between 0 g and 5 g per day	26·8%
Diet low in fibre	Average daily intake of fibre from all sources including fruits, vegetables, grains, legumes, and pulses	Consumption of fibre between 19 g and 28 g per day	88·9%
Diet suboptimal in calcium	Average daily intake of calcium from all sources, including milk, yogurt, and cheese	Consumption of calcium between 1.0 g and 1.50 g per day	88·9%
Diet low in seafood omega-3 fatty acids	Average daily intake of eicosapentaenoic acid and docosahexaenoic acid	Consumption of seafood omega-3 fatty cids between 200 mg and 300 mg per day	88·9%
Diet low in polyunsaturated fatty acids	Average daily intake of omega-6 fatty acids from all sources, mainly liquid vegetable oils, including soybean oil, corn oil, and safflower oil	Consumption of polyunsaturated fatty acids between 9% and 13% of total daily energy	88·9%
Diet high in trans fatty acids	Average daily intake of trans fat from all sources, mainly partially hydrogenated vegetable oils and ruminant products	Consumption of trans fatty acids between 0% and 1 % of total daily energy	39·9%
Diet high in sodium	24 h urinary sodium measured in mg per day	Consumption of sodium between 1 g and 5 g per day	32·3%
**Metabolic risks**			
High fasting plasma glucose	Serum fasting plasma glucose measured in mmol/L	4.8–5.4	71.2%
High total cholesterol	Serum total cholesterol, measured in mmol/L	2.78–3.38	69.2%
High systolic blood pressure	Systolic blood pressure, measured in mm Hg	110–115	79.3%
High body-mass index	Body-mass index, measured in kg/m^2	20-25	90.9%
Low glomerular filtration rate	Proportion of the population with a glomerular filtration rate <60 mL/min per 1.73 m^2, and excluding end-stage renal disease	>60 mL/min per 1.73 m2	20.2%
Low physical activity	Average weekly physical activity at work, at home, transport related, and recreational measured by metabolic-equivalent-time minute (MET min) per week	Highly active, ≥8000 MET min per week	66·7%

Data representativeness index (DRI) is the fraction of countries for which any data have been identified on the risk factor exposure.

Table S2: Data sources used for estimation of mortality and disability-adjusted life years associated with maternal and child under nutrition, low physical activity, dietary and metabolic risk factors in Ethiopia in the Global Burden of Diseases (GBD) study **(source:** http://ghdx.healthdata.org/gbd-2015-data-citations)

S. no	Data source
	**Maternal and child undernutrition**
1	Macro International, Inc, Population and Housing Census Commissions Office (PHCCO). Ethiopia Demographic and Health Survey 2005. Calverton, United States: Macro International, Inc
2	Central Statistical Agency (Ethiopia), ORC Macro. Ethiopia Demographic and Health Survey 2000. Calverton, United States: ORC Macro, 2001
3	Central Statistical Agency (Ethiopia), ICF Macro, Ministry of Health (Ethiopia). Ethiopia Demographic and Health Survey 2010-2011. Calverton, United States: ICF Macro
4	Ethiopia MICAH Program Final Evaluation Report 2006 as it appears in World Health Organization (WHO). WHO Global Database on Vitamin A Deficiency. Geneva, Switzerland: World Health Organization (WHO)
5	Haidar J, Demissie T. Malnutrition and xerophthalmia in rural communities of Ethiopia. East Afr Med J. 1999; 76(10): 590-3 as it appears in World Health Organization (WHO). WHO Global Database on Vitamin A Deficiency. Geneva, Switzerland: World Health Organization (WHO)
6	Central Statistical Agency (Ethiopia), World Bank. Ethiopia Living Standards Measurement Study - Integrated Survey on Agriculture 2011-2012. Washington DC, United States: World Bank
	Food and Agriculture Organization of the United Nations (FAO). FAOSTAT Food Balance Sheets, October 2014. Rome, Italy: Food and Agriculture Organization of the United Nations (FAO)
	**Dietary risk factors**
1	Food and Agriculture Organization of the United Nations (FAO). FAOSTAT Food Balance Sheets, May 2013. Rome, Italy: Food and Agriculture Organization of the United Nations (FAO)
2	Ethiopia - Addis Ababa STEPS Noncommunicable Disease Risk Factors Survey 2006 as it appears in Global Dietary Database Consortium, Nutrition and Chronic Disease Expert Group (NutriCoDE). Global Dietary Database 1980-2011. [Unpublished]
	**Metabolic risk factors**
1	Macro International, Inc, Population and Housing Census Commissions Office (PHCCO). Ethiopia Demographic and Health Survey 2005. Calverton, United States: Macro International, Inc
2	Central Statistical Agency (Ethiopia), ORC Macro. Ethiopia Demographic and Health Survey 2000. Calverton, United States: ORC Macro, 2001
3	Central Statistical Agency (Ethiopia), ICF Macro, Ministry of Health (Ethiopia). Ethiopia Demographic and Health Survey 2010-2011. Calverton, United States: ICF Macro
4	World Health Organization (WHO). Ethiopia World Health Survey 2003. Geneva, Switzerland: World Health Organization (WHO), 2005
5	World Health Organization (WHO). Ethiopia - Addis Ababa STEPS Noncommunicable Disease Risk Factors Survey 2006
6	World Health Organization (WHO). Ethiopia - Butajira STEPS Noncommunicable Disease Risk Factors Survey 2003
7	Tesfaye F, Nawi NG, Van Minh H, Byass P, Berhane Y, Bonita R, Wall S. Association between body mass index and blood pressure across three populations in Africa and Asia. J Hum Hypertens. 2007; 21(1): 28-37
8	Tesfaye F, Byass P, Wall S. Population based prevalence of high blood pressure among adults in Addis Ababa: uncovering a silent epidemic. BMC Cardiovasc Disord. 2009; 9(39)
9	Zein ZA, Assefa M. Blood-pressure levels and hypertension in rural Ethiopian communities. Ethiop Med J. 1986; 24(4): 169-78
10	Nshisso LD, Reese A, Gelaye B, Lemma S, Berhane Y, Williams MA. Prevalence of hypertension and diabetes among Ethiopian adults. Diabetes Metab Syndr. 2012; 6(1): 36-41
11	Abebe SM, Berhane Y, Worku A, Assefa A. Diabetes mellitus in North West Ethiopia: a community based study. BMC Public Health. 2014; 97
12	Giday A, Tadesse B. Prevalence and determinants of hypertension in rural and urban areas of southern Ethiopia. Ethiop Med J. 2011; 49(2): 139–47
13	Mengistu MD. Pattern of blood pressure distribution and prevalence of hypertension and prehypertension among adults in Northern Ethiopia: disclosing the hidden burden. BMC Cardiovasc Disord. 2014; 14: 33
14	Bonsa F, Gudina EK, Hajito KW. Prevalence of hypertension and associated factors in Bedele Town, Southwest Ethiopia. Ethiop J Health Sci. 2014; 24(1): 21–6
	**Low physical activity**
1	World Health Organization (WHO). Ethiopia World Health Survey 2003. Geneva, Switzerland: World Health Organization (WHO), 2005
2	World Health Organization (WHO). Ethiopia - Addis Ababa STEPS Noncommunicable Disease Risk Factors Survey 2006
3	World Health Organization (WHO). Ethiopia - Butajira STEPS Noncommunicable Disease Risk Factors Survey 2003

Table S3: Covariates and mediators used in Global Burden of Disease 2015 dietary risk factors study (**source:** *GBD 2015 Risk Factors Collaborators. Global, regional, and national comparative risk assessment of 79 behavioural, environmental and occupational, and metabolic risks or clusters of risks, 1990–2015: a systematic analysis for the Global Burden of Disease Study 2015. The Lancet. 2016 Oct 7; 388:1659–1724*.)

	**Covariates used in modeling**							**Metabolic mediators used**			
**Dietary risk**	**sex**	**Suboptimal metric**	**Nationally Representativeness**	**Data from FFQ**	**Data from HBS**	**Data from FAO**	**Country level covariate**	**Body Mass Index**	**Total Serum Cholesterol**	**Fasting Plasma Glucose**	**Systolic Blood Pressure**
Diet low in fruits	√	√	√	√	√	√		√	√	√	√
Diet low in vegetables	√	√	√	√	√	√		√	√	√	√
Diet low in whole grains	√	√	√	√	√	x		√	√	√	x
Diet low in nuts and seeds	√	√	√	√	√	√		√	√	√	√
Diet low in milk	√	√	√	√	√	√		x	x	x	x
Diet high in red meat	√	√	√	√	√	√		√	x	√	x
Diet high in processed meat	√	√	√	√	√	x	National availability of red meat (grams/person/day) National availability of pig meat (% of energy/person/day)	√	x	√	√
Diet high in sugar-sweetened beverages	√	√	√	√	√	x	National availability of sugar (Kcal/person/day)	x	x	x	x
Diet low in fiber	√	√	√	√	√	√		x	√	x	x
Diet suboptimal in calcium	√	√	√	√	√	√		x	x	x	x
Diet low in seafood omega-3 fatty acids	√	√	√	√	√	√	Landlocked nation (Yes,/No)	√	x	x	√
Diet low in polyunsaturated fatty acids	√	√	√	√	√	√		x	√	√	x
Diet high in trans fatty acids	√	√	√	√	√	x	National availability of hydrogenated oil (% of energy/person/day)	√	√	x	x
Diet high in sodium	√	x	√	x	x	x		x	x	x	x

FFQ – food frequency quetionaire; HBS – household budget survey; FAO-Food and Agriculture Organization; √ - used ; x – not used.

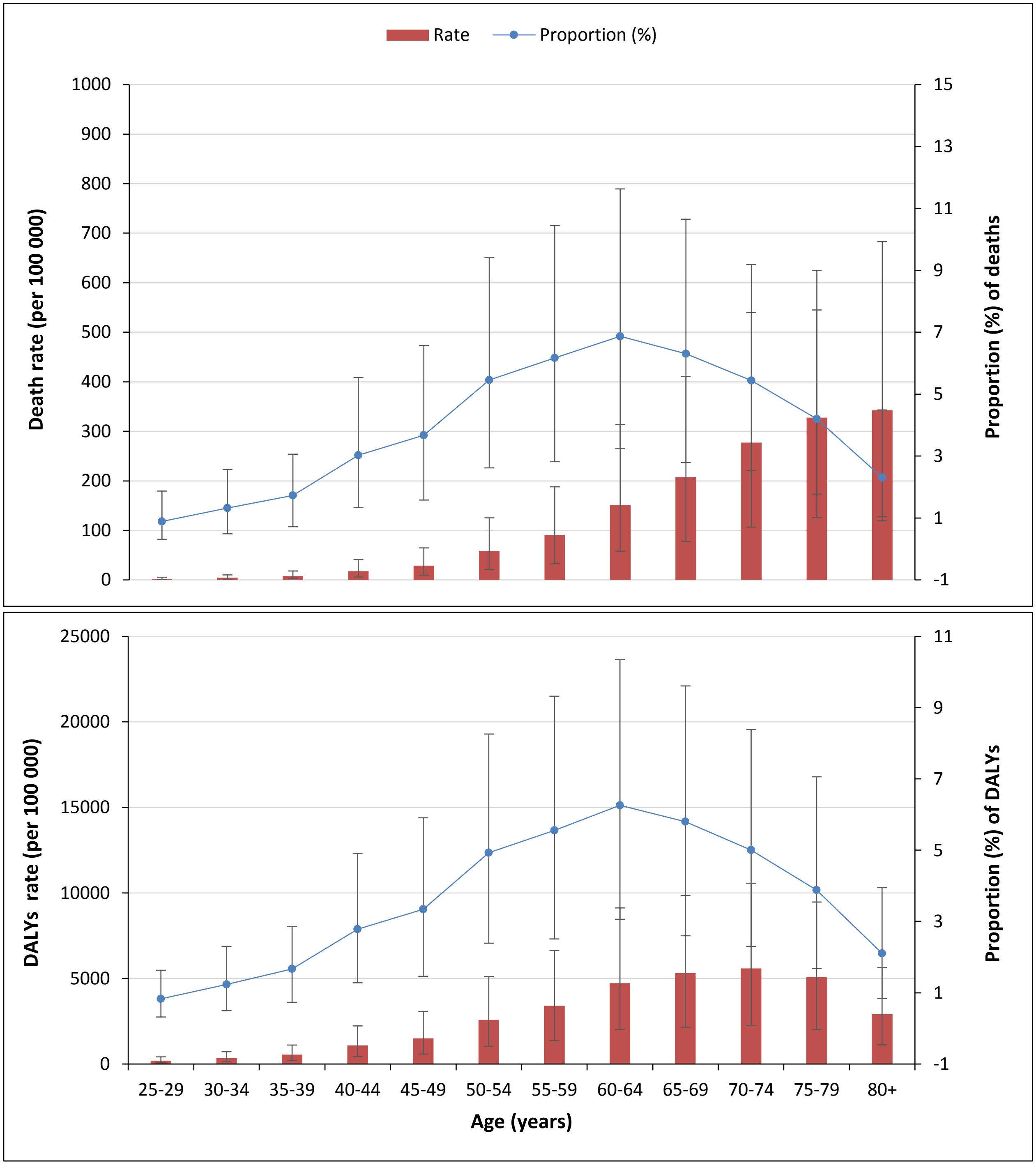

Figure S1: Rate and proportion of deaths and disability-adjusted life years (DALYs) attributable to high body mass index by age in Ethiopia, 2015 (*Proportion was calculated out of all-cause of death.*)

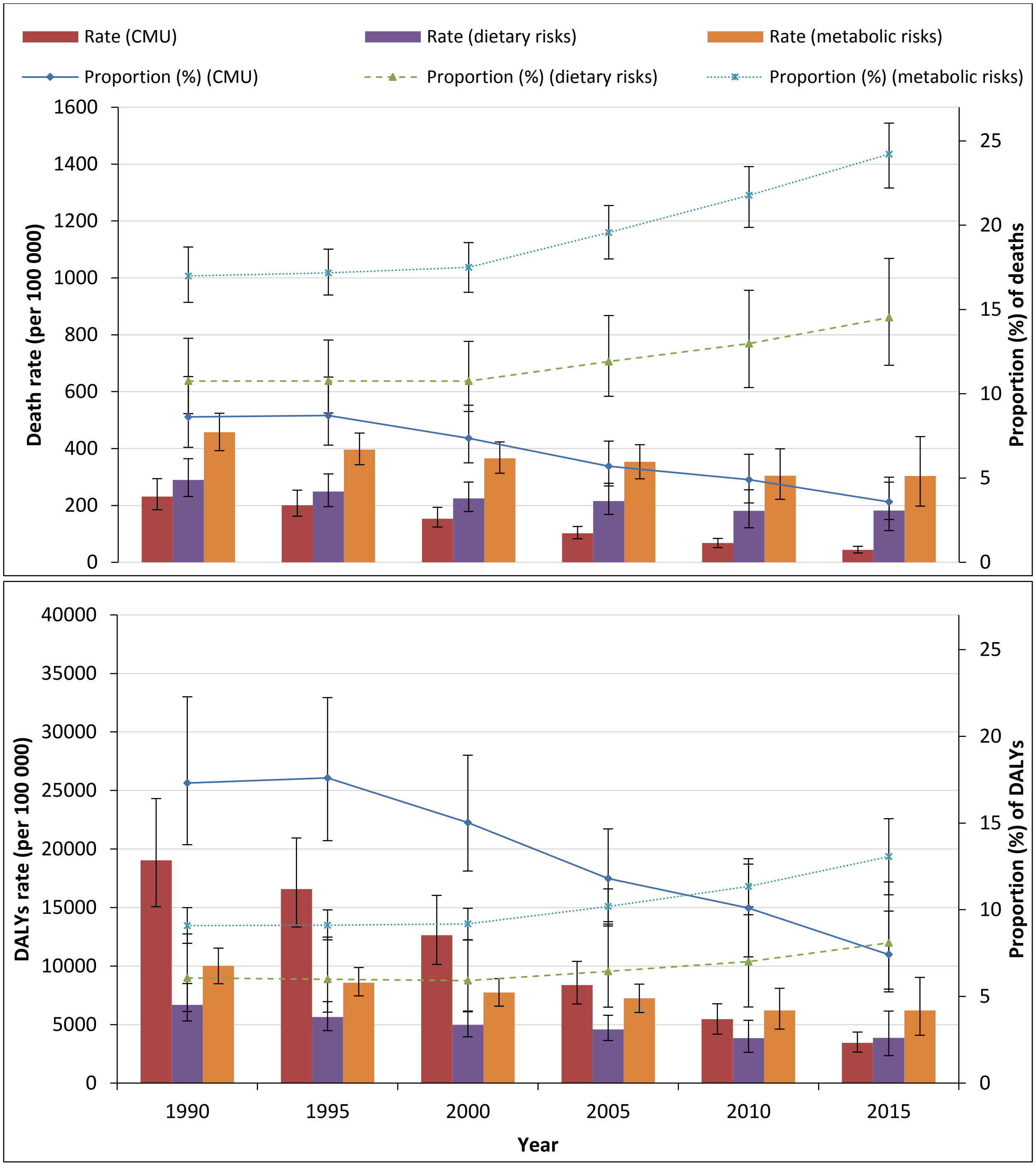

Figure S2: Trend of mortality and DALYs (age-standardized rate and proportion) attributable to child and maternal undernutrition (CMU), dietary and metabolic risks in Ethiopia, 1990-2015 (*Proportion was calculated out of all-cause of death.*)

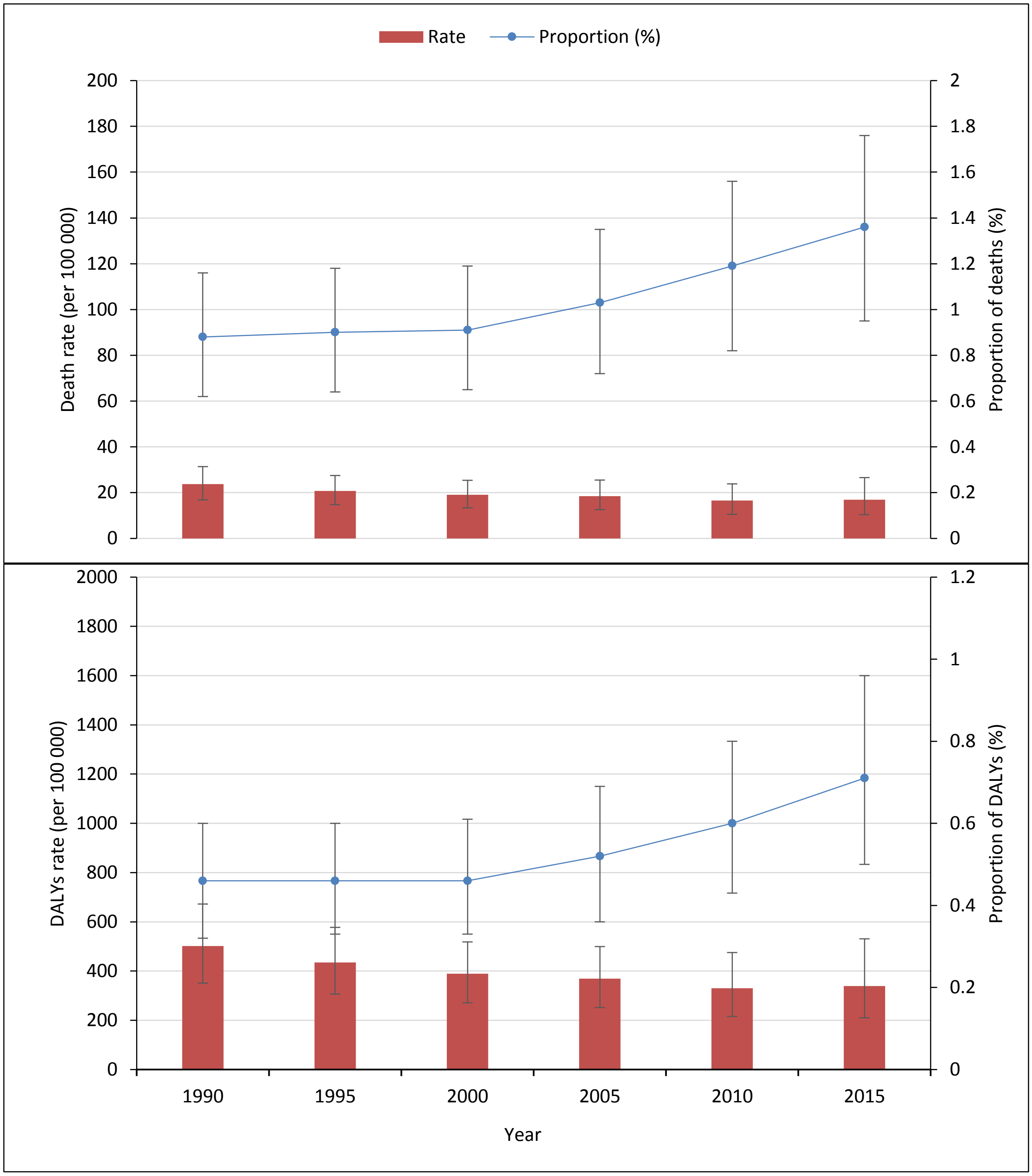

Figure S3: Trend of mortality and DALYs (age-standardized rate and proportion) attributable to low physical activity in Ethiopia, 1990-2015 (*Proportion was calculated out of all-cause of death.*)
